# A therapeutic *Porphyromonas gingivalis* gingipain vaccine induces neutralising IgG1 antibodies that protect against experimental periodontitis

**DOI:** 10.1038/npjvaccines.2016.22

**Published:** 2016-12-01

**Authors:** Neil M O’Brien-Simpson, James A Holden, Jason C Lenzo, Yan Tan, Gail C Brammar, Katrina A Walsh, William Singleton, Rebecca K H Orth, Nada Slakeski, Keith J Cross, Ivan B Darby, Dorit Becher, Tony Rowe, Adriana Baz Morelli, Andrew Hammet, Andrew Nash, Anna Brown, Bing Ma, Didier Vingadassalom, Jacqueline McCluskey, Harold Kleanthous, Eric C Reynolds

**Affiliations:** 1 Oral Health Cooperative Research Centre, Melbourne Dental School, Bio21 Institute, The University of Melbourne, Melbourne, VIC, Australia; 2 CSL Ltd., Bio21 Institute, Parkville, VIC, Australia; 3 Sanofi Pasteur, Cambridge, MA, USA

## Abstract

*Porphyromonas gingivalis* infected mice with an established *P. gingivalis*-specific inflammatory immune response were protected from developing alveolar bone resorption by therapeutic vaccination with a chimera (KAS2-A1) immunogen targeting the major virulence factors of the bacterium, the gingipain proteinases. Protection was characterised by an antigen-specific IgG1 isotype antibody and Th2 cell response. Adoptive transfer of KAS2-A1-specific IgG1 or IgG2 expressing B cells confirmed that IgG1-mediated protection. Furthermore, parenteral or intraoral administration of KAS2-A1-specific polyclonal antibodies protected against the development of *P. gingivalis*-induced bone resorption. The KAS2-A1-specific antibodies neutralised the gingipains by inhibiting: proteolytic activity, binding to host cells/proteins and co-aggregation with other periodontal bacteria. Combining key gingipain sequences into a chimera vaccine produced an effective therapeutic intervention that protected against *P. gingivalis*-induced periodontitis.

## Introduction

Chronic periodontitis is an inflammatory disease of the supporting tissues of the teeth that is associated with a polymicrobial biofilm (subgingival plaque) accreted to the tooth which results in destruction of the tooth’s supporting tissues including the alveolar bone.^1^ From epidemiological surveys moderate to severe forms of periodontitis affect one in three adults^2^ and the disease has been linked to an increased risk of cardiovascular diseases, certain cancers, preterm birth, rheumatoid arthritis and dementia related to the regular bacteremia and chronic inflammation associated with the disease.^3–7^ The global prevalence of severe periodontitis has been estimated from 2010 epidemiological data to be 10.5–12.0%^8^ and the global economic impact of dental diseases, of which periodontitis is a major component, has been estimated to be US$442 billion per year.^9^ The conventional therapy for periodontitis involves scaling and root planing to remove plaque microorganisms. Treatment can sometimes involve surgery to improve access and/or to reduce pocket depth and can also include the use of antibiotics and/or antimicrobials. However, treatment outcomes are variable and heavily dependent on patient compliance. Even in patients on a periodontal maintenance program involving regular professional intervention sites continue to progress and teeth are lost.^10^

Although chronic periodontitis is associated with a polymicrobial biofilm, specific bacterial species of the biofilm such as *Porphyromonas gingivalis*, *Treponema denticola* and *Tannerella forsythia* as a complex or consortium have been closely associated with clinical measures of disease.^11,12^ Recently the level of *P. gingivalis* in subgingival plaque above threshold levels of 10–15% of the total bacterial load has been demonstrated to predict imminent clinical attachment loss (>2 mm in 3 months) in a prospective clinical trial of chronic periodontitis patients on a clinical maintenance program.^10^ These results are consistent with previous studies showing high levels of *P. gingivalis* at refractory sites, i.e., sites where disease continued to progress after conventional scaling and root planing therapy.^13–18^

*P. gingivalis* is found at the base of deep periodontal pockets as microcolony blooms in the superficial layers of subgingival plaque adjacent to the periodontal pocket epithelium, which helps explain the strong association with underlying tissue inflammation and bone resorption at relatively low proportions (10–15%) of the total bacterial cell load in the pocket.^10,19–21^ Furthermore, it has been shown from studies using the mouse periodontitis model that *P. gingivalis* is a keystone pathogen, which dysregulates the host immune response to favour the polymicrobial biofilm disrupting homeostasis with the host to cause dysbiosis and disease.^22^ The mouse periodontitis model has also been used to show that inflammation is essential to allow establishment of *P. gingivalis* at the levels in plaque (10–15% or greater of total bacterial cell load) necessary to produce dysbiosis and alveolar bone resorption,^23^ which makes the mouse model consistent with human disease.^10,23^

The extracellular Arg- and Lys-specific proteinases ‘gingipains’ (RgpA/B and Kgp) of *P. gingivalis* have been implicated as major virulence factors that are critical for colonisation, penetration into host tissue, dysregulation of the immune response, dysbiosis and disease.^20,22,24–28^ The gingipains, in particular the Lys-specific proteinase Kgp is essential for *P. gingivalis* to induce alveolar bone resorption in the mouse periodontitis model.^29^ The gingipains have also been found in gingival tissue at sites of severe periodontitis at high concentrations proximal to the subgingival plaque and at lower concentrations at distal sites deeper into the gingival tissue.^20^ This has led to the development of a cogent mechanism to explain the keystone role played by *P. gingivalis* in the development of chronic periodontitis.^22,30^

The role of *P. gingivalis* as a keystone pathogen in the initiation and progression of chronic periodontitis suggests that a strategy of targeting the major virulence factors of the bacterium, the gingipains by vaccination may have utility in the prevention of *P. gingivalis*-induced periodontitis. Indeed, studies using the gingipains as a prophylactic vaccine that induces a high-titre antibody response in naive animals before superinfection with the pathogen have shown protection against alveolar bone resorption.^25,31,32^ However, patients with *P. gingivalis*-associated periodontitis harbour the pathogen at above threshold levels in subgingival plaque and exhibit an inflammatory immune response,^33–37^ hence it is possible that therapeutic vaccination could exacerbate inflammation and bone resorption in these patients.^38^ Here we show that therapeutic vaccination with a chimera antigen targeting the gingipains protects against alveolar bone resorption in *P. gingivalis*-associated experimental periodontitis and that this protection is mediated via a predominant Th2 anti-inflammatory response with the production of gingipain-neutralising IgG1 antibodies.

## Results

### Recombinant protein chimeras of the KAS peptide and KgpA1 domain of the RgpA–Kgp proteinase-adhesin complex are effective as prophylactic vaccines in experimental periodontitis

We have previously identified antigens from the RgpA–Kgp complex that protect against *P. gingivalis-*induced lesions and alveolar bone resorption.^25,31,39^ We hypothesised that a recombinant chimera of the most effective antigens; a Lys-gingipain active site peptide (KAS) and a recombinant Kgp-A1 adhesin sequence may exhibit enhanced protection against *P. gingivalis* induced disease by neutralising both proteolytic and adhesin functions of the virulence factor (Supplementary Figure 1). On the basis of epitope mapping and structural modelling of the Kgp proteinase and adhesin domain^25,31^ two chimera proteins were produced; KAS1-sA1 representing the minimal immunogenic sequences of KAS (Kgp-cat, 432–454) and KgpA1 (759–989) and KAS2-A1, which extended the minimal immunogenic sequences of KAS (Kgp-cat, 433–468) and KgpA1 (751–1056) (Supplementary Figure 2).

**Figure 1:**
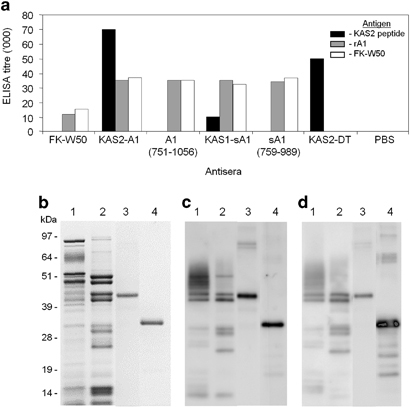
Characterisation of the antigenicity of recombinant chimera proteins KAS1-sA1 and KAS2-A1. (**a**) KAS1-sA1 and KAS2-A1 antisera were used to probe KAS2 peptide, recombinant A1 (rA1 751–1056) and formalin killed *P. gingivalis* W50 cells coated plates in an ELISA and the response compared to antisera raised to formalin killed *P. gingivalis* W50 cells (FK-W50), recombinant proteins sA1 (759–989), A1 (751–1056), KAS2 peptide-DT conjugate and PBS/IFA in an ELISA. Antibody responses are expressed as the ELISA titre OD415 obtained minus double the background level, with each titre representing the mean±s.d. of three values. (**b**) SDS-PAGE gel of; lanes: 1, *P. gingivalis* whole-cell lysate; 2, RgpA–Kgp complex; 3, KAS2-A1; and 4, KAS1-sA1. Molecular mass markers (Pharmacia) are indicated in kDa. (**c**) Western blot analysis using KAS2-A1 antisera to probe; lanes: 1, *P. gingivalis* whole-cell lysate; 2, RgpA–Kgp complex; 3, KAS2-A1; and 4, KAS1-sA1. (**d**) Western blot analysis using KAS1-sA1 antisera to probe; lanes: 1, *P. gingivalis* whole-cell lysate; 2, RgpA–Kgp complex; 3, KAS2-A1; and 4, KAS1-sA1.

Immunogenicity studies showed that KAS1-sA1 and KAS2-A1 antisera recognised KAS2 peptide, KgpA1(751–1056) and FK-*P. gingivalis* W50 cells at comparable or higher levels when compared with antisera generated to KAS2-DT, KgpA1(759–989), KgpA1(751–1056) and FK-*P. gingivalis* W50 cells (Figure 1). Only the recombinant chimera proteins were found to recognise all three (KAS2, KgpA1 and *P. gingivalis* W50 cells) antigens (Figure 1a). Western blot analysis showed that the KAS1-sA1 and KAS2-A1 antisera recognised the Kgp proteinase and adhesin domains (Figures 1b–d). Epitope mapping of the lysine proteinase active site sequence (433–468) identified that the antigenic epitopes for both KAS1 and KAS2 were around the His(444) catalytic residue^30,39^ (Supplementary Figure 3). For KAS1 the major epitopes were ^444^HGSETAWADPL^454^>^434^TGVSFANYTA^443^ and for KAS2 ^447^ETAWADPLL^455^>^434^TGVSFANYTA^443^=^456^TTSQLKAL^463^. These antigenic epitopes matched those induced by the KAS2-DT conjugate indicating that the KAS epitope in KAS1-sA1 and KAS2-A1 remained antigenic (Supplementary Figure 3).

**Figure 2:**
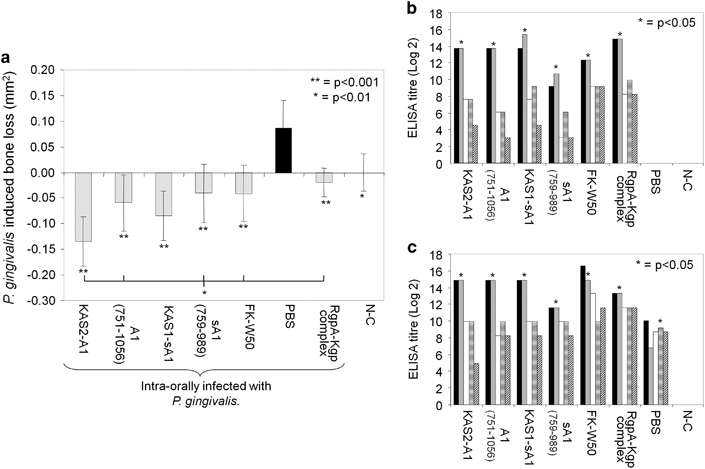
Recombinant chimera proteins KAS1-sA1 and KAS2-A1 protected against *P. gingivalis*-induced bone loss in a mouse prophylactic vaccine periodontitis model. (**a**) Mice were immunised with KAS1-sA1, KAS2-A1, RgpA–Kgp complex, formalin killed *P. gingivalis* W50 cells (FK-W50), recombinant proteins rA1 (759–989), rA1 (751–1056) or adjuvant alone (PBS, IFA). After the second immunisation mice were pre-treated with antibiotics and orally challenged with a total of eight doses of 1.0×10^10^ *P. gingivalis* W50 or treated with PG buffer containing 2% CMC alone (non-challenged group as N-C). *P. gingivalis*-induced bone resorption was determined as described in the Materials and Methods section for each group (*n*=12), and the data are expressed as the mean±s.d. in mm^2^ and were analysed using a one-way ANOVA and Dunnetts T3 *post hoc* test. * and ** indicates data that are significantly different (*P*<0.01, *P*<0.001, respectively) from the data for *P. gingivalis* challenged group. Serum antibody subclass responses of immunised mice in the periodontitis model, (**b**) post 1° and 2° immunisation and pre intra-oral challenge and (**c**) post intra-oral challenge with live *P. gingivalis* cells. Antisera was used to probe formalin killed *P. gingivalis* strain W50 as the absorbed antigen in an ELISA. Antibody responses IgG (black bars), IgG1 (grey bars), IgG2a (white bars), IgG2b (horisontal stripped bars), IgG3 (diagonal stripped bars), are expressed as the ELISA titre (log 2) obtained minus double the background level, with each titre representing the mean±s.d. of three values. * indicates IgG subclass significantly higher (*P*<0.05) than other IgG subclasses in that group.

The recombinant proteins KAS1-sA1, KAS2-A1, KgpA1(759–989), KgpA1(751–1056); FK-*P. gingivalis* W50 and the RgpA–Kgp complex were used to immunise BALB/c mice to determine their efficacy as prophylactic vaccines against *P. gingivalis-*induced alveolar bone resorption. Mice immunised with all of the recombinant antigens, RgpA–Kgp complex and FK-W50 cells exhibited significantly (*P*<0.001) less bone loss compared with that of the PBS control group (Figure 2a). Both KAS2-A1 and KAS1-sA1 immunised mice exhibited significantly less alveolar bone resorption than the mice immunised with the RgpA–Kgp complex. KAS2-A1 provided a greater level of protection (*P*<0.01) than KgpA1(759–989), KgpA1(751–1056) or FK-*P. gingivalis* W50.

Prior to intra-oral challenge with *P. gingivalis* each mouse group immunised with one of the immunogens had a high IgG antibody titre to FK-*P. gingivalis* W50 cells with a predominant IgG1 antibody subclass and only weakly immunoreactive IgG2a, IgG2b and IgG3 responses (Figure 2b). Post intra-oral challenge with *P. gingivalis* W50 each of the protective immunogens retained a predominant and significantly (*P*<0.05) higher IgG1 antibody subclass response to *P. gingivalis* (Figure 2c). However, the PBS/IFA immunised group had a predominant and significantly (*P*<0.05) higher IgG2b antibody subclass response to *P. gingivalis* (Figure 2c). Based on the above data the KAS2-A1 chimera was chosen as the lead vaccine candidate to be used in subsequent studies.

### KAS2-A1 chimera prophylactic vaccine confers protection against *P. gingivalis*-induced bone resorption at low antigen doses

**Figure 3:**
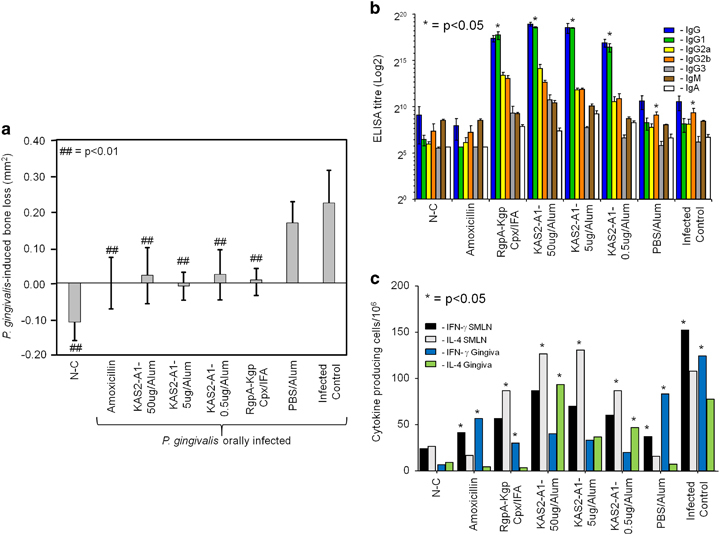
Low antigen doses of recombinant chimera protein KAS2-A1 protect against *P. gingivalis*-induced bone loss in a mouse periodontitis model. (**a**) Mice were immunised with KAS2-A1 at 50, 5.0 and 0.5 μg doses in alum, RgpA–Kgp complex/IFA, or adjuvant alone (PBS, alum). After the second immunisation mice were pre-treated with antibiotics and orally challenged with a total of eight doses of 1.0×10^10^ *P. gingivalis* W50 or treated with PG buffer containing 2% CMC alone (non-challenged group as N-C). One group of mice received amoxicillin (500 μg/ml drinking water) from the first oral challenge to the end of the experiment. *P. gingivalis*-induced bone resorption was determined as described in the Materials and Methods section for each group (*n*=12), and the data are expressed as the mean±s.d. in mm^2^ and were analysed using a one-way ANOVA and Dunnett’s T3 post-hoc test. ^##^ indicates data that are significantly different (*P*<0.01) from the data for *P. gingivalis* inoculated group. (**b**) Serum antibody subclass responses of immunised mice in the periodontitis model. Antisera was used to probe formalin killed *P. gingivalis* strain W50 as the absorbed antigen in an ELISA. Antibody responses IgG (blue bars), IgG1 (green bars), IgG2a (yellow bars), IgG2b (orange bars), IgG3 (grey bars), IgM (brown bars), IgA (white bars) are expressed as the ELISA titre (log 2) obtained minus double the background level, with each titre representing the mean±s.d. of three values. * indicates IgG subclass significantly higher (*P*<0.05) than other IgG subclasses in that group. (**c**) IFNγ and IL-4 producing submandibular lymph nodes (SMLN) T-cells and gingival tissue (gingiva) lymphocytes isolated from immunised mice in the periodontitis model. Cytokine producing cells were determined by ELISPOT using FK-*P. gingivalis* W50 as the stimulating antigen. Cytokine response expressed as cytokine producing cells/10^6^ obtained minus the background level, with data representing the mean±standard deviation of three values. * indicates which cytokine is significantly higher (*P*<0.05) than other in SMLN or gingival tissue for that group.

The KAS2-A1 chimera was formulated with aluminium hydroxide (alum) and BALB/c mice were immunised with 50 μg, 5.0 μg or 0.5 μg per dose (being adult human equivalent doses of 12, 1.2 and 0.12 mg^40^) (Figure 3). The KAS2-A1 formulated in alum at all doses trialed conferred significant (*P*<0.01) protection against *P. gingivalis*-induced bone loss (Figure 3a). There was no significant difference in the level of bone loss for the positive intervention (amoxicillin-treated), positive vaccination (RgpA–Kgp complex/IFA) and all of the KAS2-A1/alum immunised groups (Figure 3a).

Mice immunised with KAS2-A1/alum at each dose or the RgpA–Kgp complex/IFA had a high IgG titre to FK-*P. gingivalis* W50 cells with a predominant IgG1 subclass (Figure 3b). The PBS/alum immunised group and the infected control groups had a predominant and significantly (*P*<0.05) higher IgG2b antibody subclass response to *P. gingivalis* (Figure 3b).

To determine the cellular interferon (IFN)-γ and IL-4 cytokine response for each group following *P. gingivalis* oral infection, submandibular lymph node (SMLN) T cells and gingival tissue lymphocytes were isolated and the number of *P. gingivalis*-specific cytokine producing cells determined by ELISPOT (Figure 3c). The PBS/alum treated and *P. gingivalis*-infected control groups had significantly (*P*<0.05) higher numbers of IFNγ secreting SMLN T cells and gingival lymphocytes. Mice immunised with KAS2-A1/alum at all doses had significantly (*P*<0.05) higher numbers of IL-4 secreting SMLN T cells and gingival lymphocytes, characteristic of a Th2 predominant response.

### Protective efficacy of the KAS2-A1 chimera in a therapeutic vaccination periodontitis model

**Figure 4:**
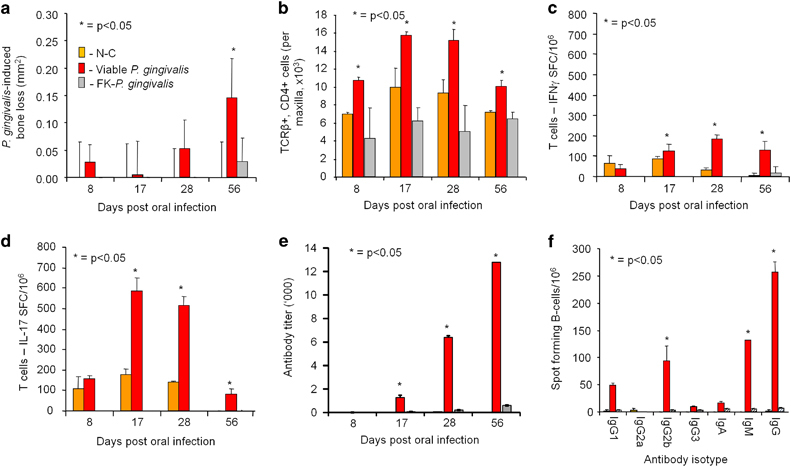
Time course analysis of the *P. gingivalis*-induced bone loss and development of a *P. gingivalis*-specific proinflammatory T-cell response. BALB/c mice were pretreated with antibiotics and then intra-orally challenged with a total of four doses of 1.0×10^10^ viable *P. gingivalis* W50 cells, formalin-killed *P. gingivalis* W50 cells (FK-*P. gingivalis*) or treated with PG buffer containing 2% CMC alone (non-challenged group N-C) on days 0, 2, 4, 6. On days 8, 17, 28 and 56 mice were killed and; (**a**) *P. gingivalis*-induced bone resorption was determined as described in the Materials and Methods section for each group (*n*=12), and the data are expressed as the mean±s.d. in mm^2^ and were analysed using a one-way ANOVA and Dunnetts T3 *post hoc* test. * indicates data that are significantly different (*P*<0.05) from the data for negative control group; (**b**) the number of gingival CD4^+^ T cells (TCRβ^+^CD4^+^) in the lymphocyte infiltrate were determined using flow cytometry using lymphocytes isolated from gingival tissue. Data are expressed as the mean±standard deviation and were analysed using Student's *t*-test. * indicates data that are significantly different (*P*<0.05) from the data for negative control group; (**c**,**d**) IFNγ and IL-17-producing submandibular lymph nodes (SMLN) T-cells were determined by ELISPOT using FK-*P. gingivalis* W50 as the absorbed antigen. Cytokine response expressed as spot forming cells per 10^6^ obtained minus the background level, with data representing the mean±s.d. of three values. * indicates which cytokine is significantly higher (*P*<0.05) from the data for negative control group; (**e**) *P. gingivalis*-specific serum antibody subclass responses were determined by ELISA using formalin killed *P. gingivalis* strain W50 as the absorbed antigen. * indicates IgG subclass significantly higher (*P*<0.05) than other IgG subclasses in that group; (**f**) *P. gingivalis*-specific antibody producing submandibular lymph nodes (SMLN) B-cells were determined by ELISPOT on day 56 using FK-*P. gingivalis* W50 as the stimulating antigen. Antibody response expressed as spot forming cells/10^6^ obtained minus the background level, with data representing the mean±s.d. of three values. * indicates Ig subclass significantly higher (*P*<0.05) than other Ig subclasses in that group.

To develop a therapeutic vaccination model we first conducted a time course study to determine the timing of a distinct *P. gingivalis*-specific immune response post intra-oral challenge (infection). Figure 4a shows that only challenge with viable *P. gingivalis* W50 cells, and not formalin-killed (FK) *P. gingivalis* cells, induced significant (*P*<0.05) bone loss. Infiltrating gingival CD4^+^ T cells were significantly (*P*<0.05) higher in numbers on days 8, 17, 28 and 56 in the viable *P. gingivalis* infected group compared with the uninfected control and the FK-*P. gingivalis* challenged group (Figure 4b). A significantly (*P*<0.05) higher number of *P. gingivalis*-specific IFNγ and IL-17 secreting T cells and *P. gingivalis*-specific serum antibody responses were found on days, 17, 28 and 56 for the viable *P. gingivalis* infected group compared with the uninfected control and the FK-*P. gingivalis* challenged group (Figures 4c–f). The time course study showed that only challenge with viable *P. gingivalis* cells induced bone loss and that an antigen-specific immune response was established from day 17 and onwards after the first oral challenge. Hence, for the therapeutic vaccination model we chose day 19 as an appropriate treatment/vaccination time point.

**Figure 5:**
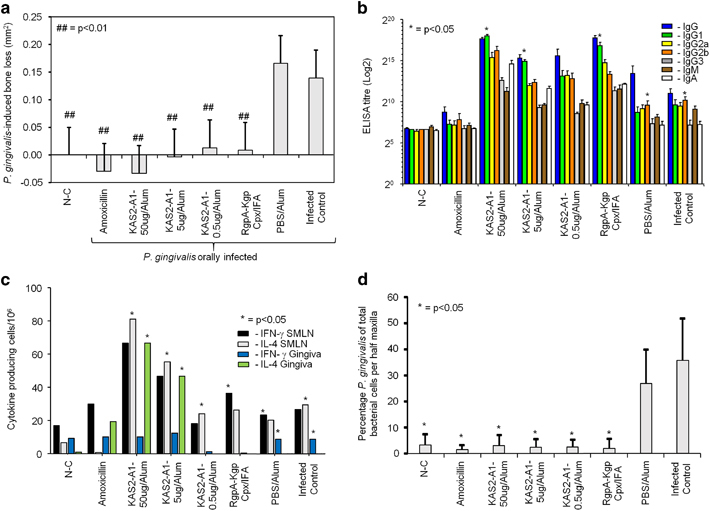
The recombinant chimera KAS2-A1 protects against *P. gingivalis*-induced bone loss in a therapeutic vaccination periodontitis model. (**a**) BALB/c mice were pre-treated with antibiotics and then orally challenged with a total of four doses of 1.0×10^10^ viable *P. gingivalis* W50 cells or treated with PG buffer containing 2% CMC alone (non-challenged group N-C) on days 0, 2, 4, 6. On day 19 after the first oral challenge, mice were immunised with KAS2-A1 at 50, 5.0 and 0.5 μg doses in alum, the RgpA–Kgp complex/IFA, or adjuvant alone (PBS, alum). A group was also treated with amoxicillin (500 μg/ml drinking water). Mice received a second immunisation (day 40) and then killed in day 62. (**a**) *P. gingivalis*-induced bone resorption was determined as described in the Materials and Methods section for each group (*n*=12), and the data are expressed as the mean±s.d. in mm^2^ and were analysed using a one-way ANOVA and Dunnetts T3 *post hoc* test. ## indicates data that are significantly different (*P*<0.01) from the data for *P. gingivalis* challenged group. (**b**) Serum antibody subclass responses of immunised mice in the periodontitis model. Antisera was used to probe formalin killed *P. gingivalis* strain W50 as the absorbed antigen in an ELISA. Antibody responses are expressed as the ELISA titre (log 2) obtained minus double the background level, with each titre representing the mean±s.d. of three values. * indicates IgG subclass significantly higher (*P*<0.05) than other IgG subclasses in that group. (**c**) IFNγ and IL-4 producing submandibular lymph nodes (SMLN) T cells and gingival tissue (gingiva) lymphocytes isolated from immunised mice in the periodontitis model. Cytokine-producing cells were determined by ELISPOT using FK-*P. gingivalis* W50 as the stimulating antigen. Cytokine response expressed as cytokine producing cells/10^6^ obtained minus the background level, with data representing the mean±s.d. of three values. * indicates which cytokine is significantly higher (*P*<0.05) than other in SMLN or gingival tissue for that group. (**d**) *P. gingivalis* cells enumerated in half maxillae from immunised mice in the periodontitis model by real time PCR, using *P. gingivalis*-specific and Universal bacteria 16S rRNA forward and reverse primers. Data are expressed as the percentage of *P. gingivalis* cells of the total bacterial cells recovered per half maxilla mean±s.d. (*n*=5) and were analysed using a Student's *t*-test. * indicates data that are significantly different (*P*<0.05) from the value for the uninoculated group.

To test the KAS2-A1 chimera as a therapeutic vaccine BALB/c mice were immunised on day 19 and 40, post oral infection with *P. gingivalis*, using the same antigen doses and alum formulation as described above. KAS2-A1-induced significant (*P*<0.01) protection against *P. gingivalis*-induced bone resorption in the therapeutic vaccination model (Figure 5a). There was no significant difference in the level of bone loss for the positive intervention (amoxicillin-treated), positive vaccination (RgpA–Kgp complex/IFA) and all of the KAS2-A1/alum immunised groups (Figure 5a).

Mice immunised with KAS2-A1/alum or the RgpA–Kgp complex/IFA had a high IgG antibody titre to FK-*P. gingivalis* cells with a predominant IgG1 antibody subclass response (Figure 5b). The PBS/alum immunised group and the infected control groups had a predominant and significantly (*P*<0.05) higher IgG2b antibody subclass response to *P. gingivalis* (Figure 5b). Mice immunised with KAS2-A1/alum at 50 and 5.0 μg doses had significantly (*P*<0.05) higher numbers of IL-4 secreting SMLN T cells and gingival lymphocytes, whereas, the PBS/alum immunised group and *P. gingivalis* infected control group had significantly (*P*<0.05) higher numbers of IFN-γ secreting SMLN T cells (PBS/alum only) and gingival lymphocytes (Figure 5c).

DNA was extracted from half maxillae of each of the groups and the *P. gingivalis* level expressed as a percentage of total bacterial cell numbers recovered. All of the KAS2-A1 immunised and RgpA–Kgp complex groups had significantly (*P*<0.05) lower levels of *P. gingivalis* as a percentage of total bacterial cells compared with the PBS/alum and infected control groups (Figure 5d).

### KAS2-A1 chimera provides protection against alveolar bone resorption in mice orally challenged with *P. gingivalis/T. denticola/T. forsythia*

**Figure 6:**
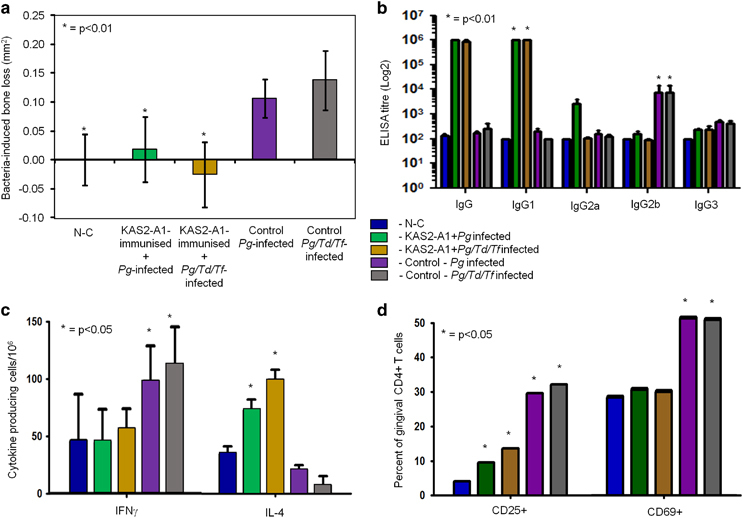
The recombinant chimera protein KAS2-A1 formulated in alum protects against bacteria-induced bone loss in a multi-pathogen superinfection periodontitis model. (**a**) Mice were immunised with KAS2-A1 at 50 μg in alum. After the second immunisation mice were pretreated with antibiotics and orally challenged with a total of four doses of 1.0×10^10^ total cells of *P. gingivalis* W50 or 1.0×10^10^ total cells of *P. gingivalis* W50/*T. denticola*/*T. forsythia* or treated with PG buffer containing 2% CMC alone (non-challenged group N-C). Bacteria-induced bone resorption was determined as described in the Materials and Methods section for each group (*n*=12), and the data are expressed as the mean±standard deviation in mm^2^ and were analysed using a one-way ANOVA and Dunnetts T3 post-hoc test. * indicates data that are significantly different (*P*<0.01) from the data for *P. gingivalis* or *P. gingivalis* W50/*T. denticola*/*T. forsythia* challenged groups. (**b**) Serum antibody subclass responses of immunised mice in the multi-pathogen periodontitis model. Antisera was used to probe formalin killed *P. gingivalis* strain W50 as the absorbed antigen in an ELISA and antibody responses are expressed as the ELISA titre (log 2) obtained minus double the background level, with each titre representing the mean±standard deviation of three values. * indicates IgG subclass significantly higher (*P*<0.01) than other IgG subclasses in that group. (**c**) IFNγ and IL-4 producing submandibular lymph node (SMLN) T-cells isolated from each group in the multi-pathogen periodontitis model. Cytokine producing cells were determined by ELISPOT using FK-*P. gingivalis* W50 as the stimulating antigen. Cytokine response expressed as cytokine producing cells/10^6^ obtained minus the background level, with data representing the mean±standard deviation of three values. * indicates which cytokine is significantly higher (*P*<0.05) than other for that group. (**d**) the per cent of gingival CD4^+^ T cells [TCRβ^+^CD4^+^] that were CD25^+^ or CD69^+^ was determined by flow cytometry using lymphocytes isolated from gingival tissue. Data are expressed as the mean±standard deviation and were analysed using Student's *t*-test. * indicates data that are significantly different (*P*<0.05) from the data for negative control group.

As *P. gingivalis* is found in subgingival plaque with *T. denticola* and *T. forsythia* we investigated whether the KAS2-A1 chimera would protect against challenge and superinfection with all three species (Figure 6). The *P. gingivalis/T. denticola/T. forsythia* challenged animals exhibited a slightly higher level of bone resorption when compared with the animals challenged with *P. gingivalis* alone at the higher dose confirming bacterial synergy in bone loss with these species.^41^ KAS2-A1 formulated in alum conferred significant (*P*<0.01) protection against *P. gingivalis/T. denticola/T. forsythia*-induced bone loss (Figure 6a). KAS2-A1 immunised animals had a predominant *P. gingivalis*-specific IgG1 (Figure 6b) and IL-4 secreting T-cell response (Figure 6c). For both *P. gingivalis/T. denticola/T. forsythia* and *P. gingivalis* infected control groups, only a *P. gingivalis*-specific antibody, IgG2b dominant (Figure 6b), and IFNγ secreting T-cell response (Figure 6c) was detected. No significant immune responses were observed towards *T. forsythia* or *T. denticola*.

Isolation and phenotyping of gingival CD69^+^ (early activated) and CD25^+^ (activated/antigen stimulated) CD4^+^ T cells showed that KAS2-A1 immunised animals had a significantly higher population of CD25^+^ but not CD69^+^, CD4^+^ gingival T cells compared with the non-infected (NC) animals. The *P. gingivalis/T. denticola/T. forsythia* and *P. gingivalis* infected control groups had a significantly higher population of CD25^+^ and CD69^+^, CD4^+^ gingival T cells compared with the non-infected and KAS2-A1 immunised animals. Taken together, the results indicate that the KAS2-A1 immunised/infected groups had developed a Th2 (IL-4 secreting) antigen-stimulated gingival T-cell population, whereas the *P. gingivalis/T. denticola/T. forsythia* and *P. gingivalis* alone infected, control groups exhibited a Th1 (INFγ secreting) early and antigen activated gingival T-cell population.

### KAS2-A1 chimera protection against *P. gingivalis*-induced bone loss is antibody mediated

**Figure 7:**
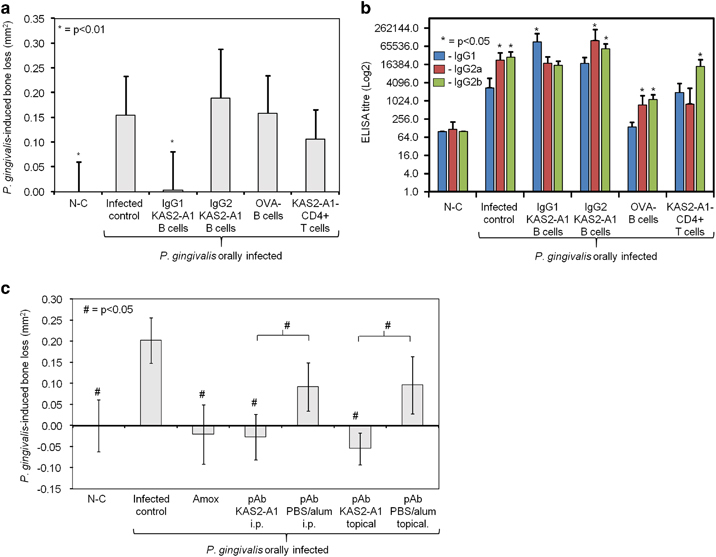
KAS2-A1 chimera protective efficacy is antibody mediate. (**a**) Adoptive transfer of IgG1 and IgG2 expressing B cells and CD4^+^ T cells from KAS2-A1 immunised mice in the periodontitis model. Donor mice (Ly5.1 C57Bl6 congenic mice) were immunised with KAS2-A1/ISCOMATRIX adjuvant (day 0) and IgG1 expressing B cells; IgG2 expressing B cells and CD4^+^ T cells isolated (day 30) from spleen and lymph nodes using magnetic bead separation and cells resuspended in PBS containing 0.5 μg of KAS2-A1/200 μl. Recipient mice (Ly5.2 C57Bl6 congenic mice) were inject (day 30) i.v. with 1×10^6^ cells; IgG1 or IgG2 expressing B cells or CD4^+^ T cells from KAS2-A1 immunised mice or B cells from ovalbumin immunised mice to act as an antigen-specific B cell control. The recipient mice had been pre-treated with antibiotics and were orally challenged (day 31) with a total of four doses of 1.0×10^10^ total cells of *P. gingivalis* W50 or treated with PG buffer containing 2% CMC alone (non-challenged group N-C). Bacteria-induced bone resorption was determined as described in the Materials and Methods section for each group (*n*=12), and the data are expressed as the mean±standard deviation in mm^2^ and were analysed using a one-way ANOVA and Dunnetts T3 post-hoc test. * indicates data that are significantly different (*P*<0.01) from the data for *P. gingivalis* infected control group. (**b**) Serum antibody subclass responses of donor mice in the periodontitis model. Antisera was used to probe formalin killed *P. gingivalis* strain W50 as the absorbed antigen in an ELISA and antibody responses are expressed as the ELISA titre (log 2) obtained minus double the background level, with each titre representing the mean±standard deviation of three values. * indicates IgG subclass significantly higher (*P*<0.05) than other IgG subclasses in that group. (**c**) Purified KAS2-A1 polyclonal antibodies administered by oral topical or intraperitoneal injection protect mice against *P. gingivalis*-induced bone loss in the periodontitis model. Purified KAS2-A1 rabbit polyclonal antibodies were injected (i.p. 500μg/mouse) one day prior oral challenge with *P. gingivalis* or applied intra-orally (topical, 2×250μg/application) 15 min pre- and post- each oral challenge. Bacteria-induced bone resorption was determined as described in the Materials and Methods section for each group (*n*=12), and the data are expressed as the mean±s.d. in mm^2^ and were analysed using a one-way ANOVA and Dunnetts T3 *post hoc* test. # indicates data that are significantly different (*P*<0.05) from the data for *P. gingivalis* infected control group.

To investigate whether KAS2-A1-specific T cells or B cells mediate protection in the mouse periodontitis model, donor mice (Ly5.1 C57Bl6 congenic mice) were immunised with KAS2-A1 chimera or ovalbumin (antigen control) and IgG1 and IgG2 expressing B cells and CD4^+^ T cells harvested and adoptively transferred into recipient mice (Ly5.2 C57Bl6 congenic mice). The day following the adoptive transfer mice were orally infected with *P. gingivalis* and bone loss determined. Mice that received CD4^+^ T cells, IgG2 expressing B cells from KAS2-A1 immunised mice or B cells from ovalbumin immunised mice developed *P. gingivalis*-induced bone loss at a similar level to that in the infected control group and had a predominant IgG2a/b response to *P. gingivalis* (Figures 7a and b). However, mice that received IgG1 expressing B cells from KAS2-A1 immunised mice did not develop *P. gingivalis*-induced bone loss and had similar alveolar bone levels to that in the non-infected (NC) control group and a predominant IgG1 response to *P. gingivalis*. Cellular phenotyping of the gingival and SMLN lymphocytes showed that mice which received IgG1 expressing B cells from KAS2-A1 immunised mice had a higher percentage of CD19^+^/CD138^+^ plasma B cells in SMLNs as well as being the only group to exhibit an increase in these cells in the gingival lymphocyte population (Supplementary Figure 4). These data indicate that B cells and not CD4^+^ T cells are pivotal for protection against *P. gingivalis*-induced bone loss and that antigen-specific IgG1, and not IgG2, is the critical IgG subclass that mediates protection.

To investigate whether KAS2-A1-specific antibodies when passively administered were able to facilitate protection against bone loss in the periodontitis model, rabbits were immunised with KAS2-A1 and polyclonal antibodies (pAb) purified using protein A/G columns. The antigenicity of the purified pAb was confirmed by ELISA and Western blot analysis (Supplementary Figure 5) and used prophylactically or therapeutically in the respective periodontitis models. In one treatment group, BALB/c mice were injected (i.p.) with KAS2-A1-pAb (500 μg per mouse) one day prior to (prophylactic model) or on day 19 post (therapeutic model) oral challenge with *P. gingivalis* to evaluate whether systemic administration could mediate protection. In a second treatment group the KAS2-A1-pAb was applied intra orally pre and post oral challenge with *P. gingivalis,* (250 μg per application, prophylactic model; or 4×500 μg doses from day 19, therapeutic model) to evaluate whether topical application of antibodies would provide protection. Both systemic and topical application of the KAS2-A1-pAb, but not control pAb, when used prophylactically or therapeutically significantly reduced *P. gingivalis*-induced bone resorption (Figure 7c; Supplementary Figure 6).

### KAS2-A1-pAb neutralisation of gingipain activities and binding to *P. gingivalis*

**Figure 8:**
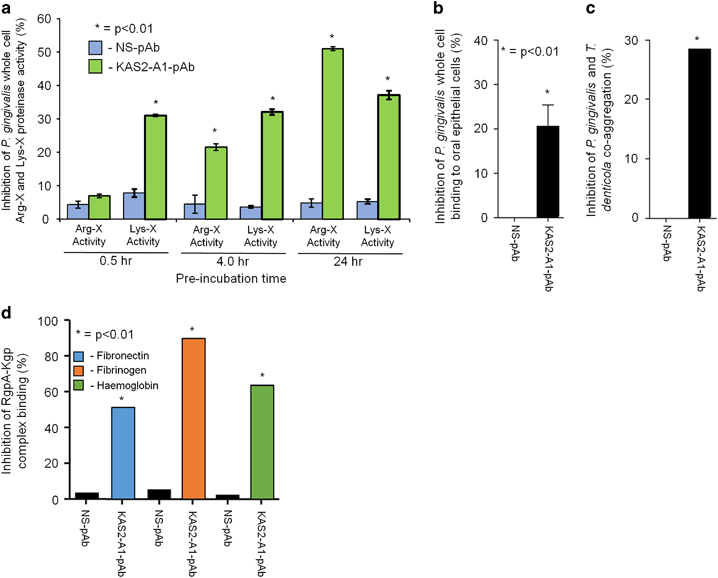
Purified KAS2-A1 pAb inhibit gingipain activities. (**a**) KAS2-A1 pAb inhibition of Lys-X and Arg-X proteinase activity of *P. gingivalis* whole cells. *P. gingivalis* W50 whole cells (1×10^8^/ml) in TC150 buffer containing 2 mmol/l L-cysteine was preincubated (0.5, 4 and 24 h) with purified KAS2-A1 pAb or non-specific (NS) pAb (100 μg/ml) and whole cell Arg-X and Lys-X proteinase activity determined. Data are expressed as the mean±s.d. of the per cent inhibition of *P. gingivalis* W50 whole cells incubated for equivalent times in the absence of antibodies and were analysed using a Student’s *T* test. * indicates data that are significantly different (*P*<0.01) from the data for the NS-pAb group. (**b**) KAS2-A1 pAb inhibition of *P. gingivalis* whole-cell binding to oral epithelial cells. FITC-labelled *P. gingivalis* W50 cells (1×10^8^/ml) in EMEM media were preincubated (60 min) with purified KAS2-A1 pAb or non-specific (NS) pAb (100 μg/ml) and then incubated (90 min) with a confluent monolayer of oral epithelial cells at a bacteria to cell ratio of 20:1. The binding of *P. gingivalis* W50 to oral epithelial cells was determined by flow cytometry and the data expressed as the mean±s.d. of the per cent inhibition of *P. gingivalis* W50 whole cells incubated for equivalent times in the absence of antibodies and were analysed using a Student’s *T* test. * indicates data that are significantly different (*P*<0.01) from the data for the NS-pAb group. (**c**) KAS2-A1 pAb inhibition of the co-aggregation of *P. gingivalis* and *T. denticola* whole cells. AlexaFluor 555-labelled *P. gingivalis* W50 cells (5×10^7^/ml) in EMEM media were pre-incubated (60 min) with purified KAS2-A1 pAb or non-specific (NS) pAb (250 μg/ml) and then incubated (60 min) with AlexaFluor 647-labelled *T. denticola* cells (5×10^7^/ml) and the level of coaggregation determined by flow cytometry. Data expressed as the mean±s.d. of the per cent inhibition of *P. gingivalis* W50 and *T. denticola* co-aggregation incubated for equivalent times in the absence of antibodies and were analysed using a Student’s *T* test. * indicates data that are significantly different (*P*<0.01) from the data for the NS-pAb group. (**d**) KAS2-A1 pAb inhibition of the RgpA–Kgp complex binding to host proteins; fibronectin, fibrinogen and haemoglobin. RgpA–Kgp complex (0.5 μg/ml) was incubated (3 h) with fibronectin, fibrinogen and haemoglobin precoated ELISA plates in the presence of KAS2-A1 pAb or non-specific (NS) pAb (5 μg/ml) in 0.1 mol/l PBS containing 1 mmol/l TLCK. The per cent of inhibition of binding was determined from the binding of RgpA–Kgp complex in the absence of antibody. Data expressed as the mean±s.d. of the per cent inhibition and were analysed using a Student’s *T* test. * indicates data that are significantly different (*P*<0.01) from the data for the NS-pAb group.

Several studies have shown that the Lys-X and Arg-X proteolytic activity, binding to oral epithelial cells, host matrix and blood proteins and co-aggregation with *T. denticola* are activities by which gingipains contribute to *P. gingivalis* virulence.^24,29,41–47^ We investigated the ability of purified KAS2-A1-pAb compared with a non-specific (NS, PBS/alum)-pAb to inhibit each of these gingipain activities associated with the bacterium’s pathogenicity (Figure 8). The KAS2-A1-pAb inhibited *P. gingivalis* whole-cell Lys-X proteolytic activity within 30 min and the level of inhibition slightly increased with incubation time (Figure 8a). Interestingly, the KAS2-A1-pAb also inhibited *P. gingivalis* whole-cell Arg-X proteolytic activity after 4 h of incubation and this increased at the 24 h time point. The ability to inhibit both Lys-X and Arg-X proteolytic activities can be attributed to the sequence similarity of the Kgp and Rgp active sites and that the minimal antigenic sequence for KAS2 (identified in Supplementary Figure 3) also occurs in the Arg-proteinases; RgpA and RgpB. KAS2-A1-pAb was also found to significantly inhibit *P. gingivalis* whole cells from binding to oral (OKF6-TERT2) epithelial cells and from co-aggregation with *T. denticola* (Figures 8b,c). The antibodies also significantly inhibited RgpA–Kgp binding to the host proteins; fibronectin, fibrinogen and haemoglobin (Figure 8d).

Further, the KAS2-A1-pAb was found to recognise all of thirteen *P. gingivalis* strains representing the major laboratory strains, serotypes A-D^43,48^ and clinical isolates (from different regions of the World) tested at a similar antibody titre when compared with RgpA–Kgp complex antibodies (Supplementary Figure 7). These data suggest that the KAS2-A1 chimera should produce antibodies that would cross-react with all currently known *P. gingivalis* strains.

## Discussion

The results of our study indicate that a chimera vaccine comprising the active site sequence (KAS) and the A1 adhesin domain of the Lys-specific gingipain Kgp when administered therapeutically switched a predominant Th1/Th17 inflammatory response to a predominant Th2 response generating gingipain-neutralising IgG1 antibodies, which protected against *P. gingivalis-*induced alveolar bone resorption. The vaccination prevented the emergence of *P. gingivalis* in subgingival plaque above threshold levels (10–15%) that cause dysbiosis and disease. The neutralising ability of the chimera-generated antibodies was confirmed as the antibodies inhibited RgpA/B and Kgp proteolytic activities, inhibited binding of the RgpA–Kgp complex to host proteins, inhibited binding to oral epithelial cells and inhibited co-aggregation with *T. denticola*. The abilities of *P. gingivalis* to adhere to oral epithelial cells and co-aggregate with *T. denticola* have been identified as key processes in colonisation and pathogenicity; and the gingipains have been shown to have a major role in these processes.^24,29,41–47^ The neutralisation of gingipain activity would help explain the protective mechanism of the antibodies in terms of preventing alveolar bone resorption as the gingipains are essential for *P. gingivalis* virulence and have been shown to be critical for colonisation, penetration into host tissue, dysregulation of the immune response, chronic inflammation, dysbiosis and alveolar bone resorption.^20,22,24–28^ The protection conferred by the chimera vaccine was also observed in the mouse periodontitis model infected with *P. gingivalis*, *T. denticola* and *T. forsythia* confirming the key pathogenic role of *P. gingivalis.* Recently, the targeting of keystone bacterial pathogens in the diverse microbial communities of saliva and the gut has been shown to not only result in the reduction in the level of the keystone pathogen, but also in the level of synergistic accessory pathogens and the promotion and restoration of commensal species and homeostasis.^49–51^ The KAS-A1 chimera-specific antibodies recognised all of the *P. gingivalis* serotypes, laboratory-type strains and clinical isolates tested, indicating high conservation of the gingipain active site and A1 adhesin sequences.

The protection conferred by the chimera vaccine was mediated by the gingipain-neutralising IgG1 antibodies generated as shown by the adoptive transfer and passive administration (systemic and topical) experiments. An interesting result from these experiments was that the KAS2-A1 specific IgG2-expressing B cells were not protective and did not home to the SMLN or gingiva as effectively as the IgG1 expressing B cells. Also, the adoptive transfer of the KAS2-A1 stimulated CD4^+^ T cells did not protect mice from *P. gingivalis* induced bone resorption indicating that antigen-specific T helper cells alone are insufficient to stimulate production of specific gingipain antibodies and induce protection. Th cells are nevertheless important in periodontitis as a protective response to the chimera vaccine was characterised by a switch from a Th1/Th17- to a Th2-biased response. This is consistent with previous studies in mice and humans where periodontitis progression has been associated with a predominant Th1/Th17 response and health/stability has been associated with a predominant Th2 response.^33–37,52^ In fact, Moutsopoulos *et al.*^34^ have shown that *P. gingivalis*, facilitated by the gingipain proteinases induced Th17 cell activation and that these cells may have a major role in orchestrating chronic inflammation. Our current data showing that Th17 cells are a major T-cell subset at the onset of *P. gingivalis*-induced disease in mice corroborates these findings. Hence, the ability of anti-KAS2-A1 antibodies to inhibit *P. gingivalis* proteinase activity would help reduce the activation of Th17 cells; thus ameliorating the inflammatory immune response orchestrated by these cells.

T helper cells also have an important role as the Th immune bias does effect antibody class switching as antigen-stimulated B cells secrete IgG1 or IgG2a/b/G3 subclasses in the presence of IL-4 or IFNγ, respectively.^53,54^ Antibody subclasses are known to contribute to the progression of the immune response to a pathogen and have substantial impacts on immunotherapeutic or immunopathogenic outcomes.^55,56^ Phagocytosis of murine IgG1 opsonised pathogens induces anti-inflammatory cytokine/chemokine secretion, whereas, IgG2a/b/G3 opsonised pathogens stimulate an inflammatory cytokine/chemokine response upon phagocytosis.^53,54^ We have previously shown in humans with a high IgG4 (mouse IgG1 equivalent) response to *P. gingivalis* that they are periodontally stable and recognise epitopes in the KgpA1 adhesin domain that are not recognised by periodontitis patients with progressive disease who have a high IgG2 (mouse IgG2a/b equivalent) response to *P. gingivalis* LPS.^33^ These A1 adhesin epitopes (EP1–3 and ABM1–3, Supplementary Figure 1) have been shown to be protective and are incorporated into the chimera vaccine.

The KAS2-A1 chimera formulated in a regulatory approved adjuvant ‘alum’ was an effective therapeutic vaccine against the induction of periodontal bone loss in the mouse model suggesting that it may have utility in the adjunctive treatment to scaling and root planing for patients with chronic periodontitis who have a pre-existing immune response to *P. gingivalis*. Several clinical studies in humans support the concept of vaccination with the gingipains and the generation of protective antibodies to prevent *P. gingivalis* emergence in plaque and progression of periodontitis.^33,57,58^ Booth *et al.*^57^ showed that subgingival application of an anti-gingipain A1 adhesin monoclonal antibody could prevent recolonisation of subgingival plaque by *P. gingivalis*. This result was confirmed by Yokokama *et al.*^58^ using anti-gingipain antibodies who showed a reduction in the plaque levels of *P. gingivalis* and also a significant reduction in pocket depth and bleeding on probing in the periodontitis patients who received the specific antibodies. O’Brien-Simpson *et al.*^33^ showed that subjects who had naturally developed a specific IgG4 response to the gingipains did not exhibit progressive disease and appeared stable compared with those subjects with predominant IgG2/IgG3 responses. These results are consistent with periodontitis in humans being associated with an inflammatory Th1/Th17 response (high IgG3/IgG2), whereas periodontal health/stability is more associated with an anti-inflammatory Th2 response (high IgG4/IgG1) as discussed above.

In conclusion, the results suggest that vaccination of humans with chronic periodontitis using the *P. gingivalis* gingipain chimera as an adjunct to scaling and root planing should induce a switch to a Th2 (less inflammatory) response generating gingipain neutralising antibodies that should help prevent re-emergence of *P. gingivalis* in subgingival plaque and thereby prevent dysbiosis and disease progression. As chronic periodontitis is a known risk factor for cardiovascular diseases, diabetes, spontaneous preterm birth and low-birth-weight infants, pancreatic cancer and rheumatoid arthritis^59–63^ the vaccine may also have broader health benefits.

## Materials and Methods

All Materials and Methods are provided in the Supplementary Information.
