# Supplementary material for: A therapeutic Porphyromonas gingivalis gingipain vaccine induces neutralising IgG1 antibodies that protect against experimental periodontitis
Source: NPJ Vaccines. 2016 Dec 1;1:16022–. doi: 10.1038/npjvaccines.2016.22 (PMC5707886; doi:10.1038/npjvaccines.2016.22)
Supplement: Supplementary Information [file npjvaccines201622-s1.doc]

**SUPPLEMENTARY INFORMATION**

**MATERIALS AND METHODS**

**Bacterial strains and growth conditions**

*P. gingivalis* strains; W50 (serotype C); 381 (serotype A), A7A1-28 (serotype B), ATCC 33277 (serotype D), ATCC 53978, ATCC 49417, YH522 and clinical isolates 84-3, RA, 3-3, 3A1, 7B-TORR and 15-9 were obtained from the culture collection of the Oral Health Cooperative Research Centre, The Melbourne Dental School, University of Melbourne, Australia. *P. gingivalis* strains were grown on Horse Blood Agar (HBA) (20 g/L HBA; Oxoid Ltd., Hampshire, UK) supplemented with 10% v/v lysed horse blood (37 °C) in an anaerobic N_2_ atmosphere containing 5% CO_2_ in a MK3 Anaerobic Workstation (Don Whitley Scientific Ltd., Adelaide, Australia). Colonies were inoculated into starter culture comprised of 20 mL sterilised brain heart infusion (37 g/L BHI; Oxoid Ltd., Hamsphire, UK) medium supplemented with 5 mg/L hemin and 0.5 mg/L cysteine[^1^](#_ENREF_1) and incubated anaerobically (24 h, 37 °C). Absorbance of batch cultures were monitored at OD_650nm_ using a spectrophotometer (model 295E, Perkin-Elmer, Germany). Bacterial cells were harvested during late exponential growth by centrifugation (7,000 *g*, 20 min, 4 °C). Bacterial purity was routinely confirmed by Gram stain[^2^](#_ENREF_2).

*Treponema denticola* ATCC 35405 was obtained from the culture collection of the Oral Health CRC, The University of Melbourne. *T. denticola* ATCC 35405[^3^](#_ENREF_3) was grown in oral bacterial growth medium (OBGM), a modified version of NOS[^4^](#_ENREF_4) and GM-1[^5^](#_ENREF_5) media containing; brain heart infusion (12.5 g/L), trypticase (10 g/L), yeast extract (7.5 g/L), sodium thioglycolate (0.5 g/L), asparagine (0.25 g/L) D-glucose (2 g/L), ascorbic acid (2 g/L), pyruvic acid (1 g/L) and sodium chloride (2 g/L). The medium was supplemented with cysteine (1 g/L), ammonium sulfate (2 g/L), thiamine pyrophosphate (6 mg/L), sodium hydrogen carbonate (2 g/L), heat inactivated rabbit serum (2% vol/vol), haemin (5 mg/L), menadione (1 mg/L) and volatile fatty acid mix (0.5% vol/vol). The volatile fatty acid mix was 0.1 M potassium hydroxide containing isobutyric acid (0.5% vol/vol), DL-2-methylbutyric acid (0.5% vol/vol), isovaleric acid (0.5% vol/vol), valeric acid (0.5% vol/vol). All chemicals were supplied by Sigma, and the growth media was supplied by Oxoid. *T. denticola* was grown in continuous culture using either a model C-30 BioFlo chemostat (New Brunswick Scientific, USA) with a working volume of 365 mL or a BioFlo 110 Modular Benchtop Fermentor (New Brunswick Scientific) with a working volume of 900 mL. The bacteria were grown at 37°C with constant agitation (30 rpm) under a constant stream of anaerobic gas (5% CO_2_ and 4% H_2_ in N_2_). The dilution rate was 0.044 h^-1^ giving a mean generation time of 15.75 h and the pH was maintained at 7.4 ± 0.1. Bacterial cell density was monitored using spectrophotometry at a wavelength of 650 nm (AU_650_; Novaspec III, Amersham Biosciences). Culture purity was determined daily by Gram stain. Bacterial cell culture samples were collected aseptically from the chemostat overflow.

*Tannerella forsythia* ATCC 43037 was obtained from the culture collection of the Oral Health CRC, The University of Melbourne *T. forsythia* was cultured as previously described[^6^](#_ENREF_6) in Trypticase soy (15 g/liter), BHI (18.5 g/liter) supplemented with yeast extract (10 g/liter), hemin (5 mg/liter), menadione (0.4 mg/liter), *N*-acetyl muramic acid (10 mg/liter), cysteine (0.5 g/liter), and fetal bovine serum (5% [vol/vol]). Absorbance of batch cultures were monitored at OD_650nm_ using a spectrophotometer (model 295E, Perkin-Elmer, Germany). Bacterial cells were harvested during late exponential growth by centrifugation (7,000 *g*, 20 min, 4 °C). Bacterial purity was routinely confirmed by Gram stain[^2^](#_ENREF_2).

**Bacteria enumeration**

Bacterial cells were enumerated using the Molecular Probes Bacteria Counting Kit using the manufacturer’s protocol where dilutions of the bacterial cells were performed in media (5% w/v trypticase soy broth (TSB) in 0.15 M NaCl). In order to create a standard curve, bacterial cultures of various cell densities as determined by measuring absorbance at a wavelength of 650 nm were used. Where multiple dilutions were examined, the highest cell density of bacteria used was ~1 x 10^6^ cells/mL. Aliquots of 10 μL of these dilutions were mixed with 990 μL cytometry media. Modifications to the manufacturer’s protocol included the addition of 10 μL of the microsphere suspension, to give a final concentration of 1 x 10^6^ microspheres/mL, to each sample before the addition of the provided SYTO BC bacterial stain. Also differing from the standard protocol, the 1 μL of SYTO BC added was first diluted 1 in 2 in the media. Controls used in this experiment were samples containing bacterial cells with SYTO BC stain but no microspheres, bacterial cells with microspheres but no SYTO BC stain, and microspheres with SYTO BC stain but no bacteria. Flow cytometry analysis was carried out on a Beckman Coulter Cytomics FC 500 cytometer equipped with a Uniphase Argon ion laser set at 488 nm with a 20 mV output.

**Preparation of formalin-killed *P. gingivalis* W50 (FK W50)**

*P. gingivalis* W50 culture was harvested (6,500 *g*, 4 ºC), washed once with phosphate buffered saline (PBS) (0.01 M Na_2_HPO_4_, 1.5 mM KH_2_PO_4_ and 0.15 M NaCl, pH 7.4) then pelleted by centrifugation (7,000 *g,* 20 min 4 ºC). Bacterial cells were resuspended (gentle shake, 24 h, 22 °C) in 0.5% v/v formal saline on a rocking platform (model RSM6, RATEK Instrument Pty Ltd, Australia). The suspension was centrifuged (7,000 *g,* 20 min 4 ºC) and resuspended in sterile PBS and this was repeated once. After the second wash, the supernatant was discarded and the cell pellet was resuspended in sterile PBS to obtain a cell density of 2 x 10^10^ cells/mL, and protein concentration determined using Biorad Protein Assay Dye Reagent Concentrate (Life Science, NSW, Australia).

**Preparation of *P. gingivalis* membrane extract**

Preparation of *P. gingivalis* membrane extracts was similar to that described previously was used[^7^](#_ENREF_7). *P. gingivalis* strains; W50 (serotype C); 381 (serotype A), A7A1-28 (serotype B), ATCC 33277 (serotype D), ATCC 53978, ATCC 49417, YH522 and clinical isolates 84-3, RA, 3-3, 3A1, 7B-TORR and 15-9 were cultured and cells harvested (6,500 *g*, 4 ºC), washed once with TC 50 buffer (50 mM sodium chloride, 50 mM Tris, 5 mM calcium chloride, pH 7.4) then pelleted by centrifugation (7,000 *g,* 20 min 4 ºC). The pellet was then suspended in TC 50 buffer and 0.5% TX-114 (v/v) added, which was followed by gentle agitation at room temperature for 60 min. The solution was centrifuged (10,000 *g*, 30 min) and the supernatant collected and immediately stored at -20^o^C until used.

**Purification of the RgpA-Kgp proteinase-adhesin complexes (RgpA-Kgp complexes)**

The protein extraction and purification of the RgpA-Kgp complexes (from strain W50) was performed as described by Pathirana *et al.*[^7^](#_ENREF_7).

**Construction of pET28 constructs containing adhesin sequences and adhesin sequences with N-terminal addition of Kgp proteinase sequences**

Kgp residues representing peptides and chimeric peptides of the active site (KAS) and KgpA1 adhesin (A1) domains were over-expressed in *E. coli* as recombinant (r) proteins with hexa-His tags using pET expression vectors (Novagen). The r-proteins expressed were rKAS2, and rA1 and the r-chimeric proteins were KAS2-A1 and KAS1-sA1. The amino acid sequences representing the various A1 and KAS domains are described in **Fig. 2**.

The various KAS and A1 domains of the *kgp* gene were amplified from pNS1 (3.5 kb BamHI *lys* fragment in pUC18) or *P. gingivalis* genomic DNA respectively using primers listed in **Supplementary** **Table 1.** Taq DNA polymerase (Invitrogen) and a PC‑960 thermal cycler (Corbett Research Technologies). Primer pairs KAS2-FOR and KAS2-REV and A1-FOR and A1-REV were used to generate PCR fragments encoding KAS2 and A1 respectively using the following reaction conditions: 94°C, 3 minutes, followed by 28 cycles of 94°C, 45 sec (denaturing); 62°C, 40 seconds (annealing) and 72°C, 20 seconds (extension) followed by a final cycle of 72°C, 5 min.

The KAS2-A1 chimeric PCR product was produced by gene splicing by overlap extension (SOEing) as follows: PCR products were produced using primer pairs KAS2-FOR and KAS2-A1-REV and KAS2-A1-FOR and A1-REV using the conditions described above. The PCR products were then annealed and a final PCR was performed with primers KAS2-FOR and A1-REV (94°C, 2 minutes, followed by 28 cycles of 94°C, 30 sec; 50°C, 30 seconds and 72°C, 40 seconds followed by a final cycle of 72°C, 5 min.

For the preparation of the KAS1-sA1 PCR product, two successive PCR’s were conducted using the KAS1-sA1-REV primer with each of the KAS1-sA1-FOR primers 1 and 2 in succession (reaction conditions 94°C for 2 minutes followed by 35 cycles of 94°C, 15 seconds ; 63°C, 30 seconds and 72°C, 2 minutes) to produce the KAS1-sA1 PCR product. The KAS1-sA1-FOR1 and KAS1-sA1-FOR2 primers contain an 3’extension overlapping the 5’ of the previous PCR product.

All of the PCR fragments encoding KAS2, A1, KAS2-A1 and KAS1-sA1 were purified using PCR purification columns (Qiagen), ligated into the TA cloning vector, pGem-T Easy (Promega) and transformed into *E. coli* JM109 following the manufacturer’s protocol. Purified recombinant pGemT-Easy constructs were digested with NcoI and XhoI and directionally cloned into NcoI/XhoI digested pET28b (Novagen) and transformed into the non-expression host, *E. coli* JM109 [DH5α]. The recombinant pET28 constructs were purified and transformed into the *E. coli* expression host, BL21 (DE3) [HMS174(DE3)] (Novagen) and selected on LB containing 50 μg kanamycin following the manufacturer’s instructions. The integrity of each insert was confirmed by DNA sequence analysis.

The oligonucleotide primers (**Supplementary Table 1**) have been designed to incorporate restriction enzyme sites, stop codons and hexa-His Tags where necessary. The primers used for the KAS2, A1 and KAS2-A1 were designed to limit the inclusion of extraneous coding sequence to no more than three amino acids plus the hexa-his tag in r-proteins. The KAS1 and the A1 were designed to contain a hexa-His tag at the N-terminal and C-terminal ends respectively, so that they may be directly compared to the KAS2-A1 which has a hexa-his tag at both N- and C-termini. In KAS1-sA1 the His Tags are found at the C-termini.

**Expression and purification of recombinant proteins**

Recombinant proteins were expressed from pET28:: A1(KAS2, KAS2-A1, KAS1-sA1) constructs by induction with isopropyl β-D-thiogalactosidase (IPTG). All recombinant proteins were produced as 6-His Tag fusion proteins and purified with NI-NTA purification system (Invitrogen) under denaturing conditions as previously described[^8^](#_ENREF_8). Briefly, *E. coli* (DE3) single colony transformants were used to inoculate 20 mL of Luria‑Bertani (LB) broth containing 50 μg/ml kanamycin at 37°C on an orbital shaker overnight. This inoculum was then used to inoculate 1L of LB containing 50 μg/ml kanamycin. The OD_600_ of this culture was allowed to reach 0.5-0.7 (mid-log phase) before inducing protein expression with isopropyl IPTG at 1.0 mM for 2 hours at 37°C with shaking of 200 rpm. Cells were harvested (7,500g) and resuspended in a denaturing binding buffer (8M Urea, 20 mM Sodium Phosphate pH 8.0 & 500 mM NaCl) and sonicated on ice for 3 x 15 s bursts at 30 s intervals using a Branson Sonifer 250 Cell disrupter (Branson Ultronics Corporation, Danbury, CT) with the microtip on setting 3, then centrifuged at 39,000 g for 30 min at 4°C. Recombinant proteins were purified from the supernatant by loading onto a pre-equilibrated Ni-NTA Agarose column and then washing with denaturing washing buffer (8M Urea, 20 mM Sodium Phosphate pH 6.0 & 500 mM NaCl) to elute unbound proteins. The column was then washed using 10 volumes of binding buffer B and the recombinant protein was eluted with denaturing elution buffer (8M Urea, 20mM Sodium Phosphate pH 6.0, 500mM NaCl & 0.5 M Imidazole). Purified protein was dialyzed against 2M Urea-PBS and stored at -80^o^C. Recombinant sA1 (759-989) and A1 (751-1056) were produced as previously described[^8^](#_ENREF_8).

Recombinant protein samples were analyzed by SDS-PAGE and their molecular masses determined using ProtParam on-line (<http://au.expasy.org/tools/protparam.html>). Protein concentration of all samples was determined by the Bio-Rad Protein Assay using BSA as a standard.

**SDS-PAGE gel electrophoresis and Western blotting**

Recombinant proteins (10 μg) were analyzed using the XCell surelock Mini-Cell electrophoresis system. Recombinant proteins were mixed in 20 µl of reducing sample buffer (10% [wt/vol] SDS, 0.05% [wt/vol] bromophenol blue, 25% [vol/vol] glycerol, and 0.05% [vol/vol] 2-mercaptoethanol). The pH was adjusted to pH 8.0 with 1.5 M Tris-HCl, and then the solution was heated for 5 min at 100°C. Recombinant proteins (10 µg/lane) were loaded onto Novex 12% (wt/vol) Tris-glycine precast mini gels, and electrophoresis was performed using a current of 30 to 50 mA and a potential difference of 125 V using a Novex electrophoresis system (Novex, San Diego, CA). Proteins were visualized using 0.25% w/v Coomassie blue R250.

**Animal ethics**

All animal experimental procedures were carried out in strict accordance with the recommendations in the *Australian Code of Practice for the Care and Use of Animals for Scientific Purposes.* The protocols for the experiments were approved by The University of Melbourne Ethics Committee for Animal Experimentation (Approval Number 081049).

**Mouse periodontitis model**

All animal experiments were approved by The University of Melbourne Ethics Committee for Animal Experimentation. The mouse periodontitis experiments were modified from Baker et al.'s model[^9^](#_ENREF_9), and performed as described previously by O’Brien-Simpson *et al.*[^10^](#_ENREF_10). The number of mice per group was based on power estimates from previous work[^10^](#_ENREF_10) and mice were randomly allocated to each group. Mice (female BALB/c or C57BL/6;, 6-8 weeks old, 12 mice/group), were given, on day -7, kanamycin (Sigma-Aldrich, New South Wales, Australia) (1 mg/mL) in deionized water *ad libitum* for seven days followed by a three days antibiotic-free period. On Day 0, mice were intra-orally challenged with *P. gingivalis* consisting of four doses of *P. gingivalis* W50 [1 x 10^10^ viable *P. gingivalis* W50 cells suspended in 20 µL PG buffer (50 mM Tris-HCL, 150 mM NaCl, 10 mM MgSO_4_ and 14.3 mM Mercaptoethanol, pH 7.4) containing 2% w/v carboxymethylcellulose (CMC, Sigma, New South Wales, Australia)], given two days apart. The bacterial challenge was prepared anaerobically and then immediately applied to the gingival margin of the maxillary molar teeth. The number of viable bacteria in each inoculum (challenge) was verified by flow cytometry and CFU counts on blood agar. On Day 58, mice were bled and killed. Maxillae were removed and halved through the midline, with 12 halves used to determine alveolar bone loss, 6 halves used to enumerate *P. gingivalis* W50 colonization of gingival plaque by real-time PCR and 6 halves used for flow cytometric analysis or used for ELISPOT studies. Submandibular lymph nodes were also removed for ELISPOT analysis. Sera were used to determine the antibody profile using ELISA. Maxillae, tissue, plaque and sera samples were coded and analyzed/examined by personnel blinded to the code. The code was only released after all the analytic data were entered.

For the prophylactic vaccination experiments; BALB/c mice were immunized with 50μg/dose of either KAS1-sA1, KAS2-A1, the RgpA-Kgp complex, formalin killed *P. gingivalis* W50 cells (FK-W50), recombinant proteins rA1 (759-989) or rA1 (751-1056), one group was immunized with adjuvant alone (PBS, IFA) or not infected (non- challenged control, N-C) mice. After thirty days mice received a second immunization and four days later were treated with antibiotics and orally challenged with *P. gingivalis* W50 (Day 0, four doses of *P. gingivalis* W50 [1 x 10^10^ viable *P. gingivalis* W50 cells suspended in 20 µL PG buffer containing 2% w/v CMC], given two days apart. Mice received a second intra-oral challenge (day 21) as described above) and alveolar bone loss determined and bio-assays preformed as described above.

For the prophylactic KAS2-A1 vaccine dosing experiments; BALB/c mice were immunized with KAS2-A1 at 50 μg, 5.0 μg and 0.5 μg doses in alum, the RgpA-Kgp complex/IFA, one group was immunized with adjuvant alone (PBS, IFA) or not infected (non-challenged control, N-C) mice. After thirty days mice received a second immunization and four days later were treated with antibiotics and orally challenged with *P. gingivalis* W50 (Day 0, four doses of *P. gingivalis* W50 [1 x 10^10^ viable *P. gingivalis* W50 cells suspended in 20 µL PG buffer containing 2% w/v CMC], given two days apart. Mice received a second intra-oral challenge (day 21) as described above) and alveolar bone loss determined and bio-assays preformed as described above.

For the time course experiments; BALB/c mice were pre-treated with antibiotics and then orally challenged with a total of four doses of 1.0 x 10^10^ viable *P. gingivalis* W50 cells; 1.0 x 10^10^ FK-*P. gingivalis* W50 cells or treated with PG buffer containing 2 % CMC alone (non-challenged group, N-C) on days 0, 2, 4, 6. On days 8, 17, 28 and 56 mice were killed and alveolar bone loss determined and bio-assays preformed as described above.

For the therapeutic vaccination experiments; BALB/c mice were pre-treated with antibiotics and then orally challenged with a total of four doses of 1.0 x 10^10^ viable *P. gingivalis* W50 cells or treated with PG buffer containing 2 % CMC alone (non-challenged group, N-C) on days 0, 2, 4, 6 as described above. On day 19 after the first oral challenge, mice were then immunized with KAS2-A1 at 50 μg, 5.0 μg and 0.5 μg doses in alum, KAS2-A1 at 50 μg in PBS, 50μg RgpA-Kgp complex in IFA, adjuvant alone (PBS, alum), treated with amoxicillin (500µg/mL drinking water) or not infected (non-challenged, N-C) mice. Mice received a second immunization (day 40) and then killed in day 62 and alveolar bone loss determined and bio-assays preformed as described above.

For the multi-pathogen experiments; BALB/c mice were immunized (day 0) with KAS2-A1 at 50 μg in alum. After thirty days mice received a second immunization and four days later were treated with antibiotics and orally challenged with a total of four doses of 1.0 x 10^10^ total cells of *P. gingivalis* W50 or 1.0 x 10^10^ total cells of a co-challenge of *P. gingivalis* W50/*T. denticola*/*T. forsythia* or treated with PG buffer containing 2 % CMC alone (non-challenged group as N-C). Mice were killed 62 days from the first oral challenge and alveolar bone loss determined and bio-assays preformed as described above.

For the cell adoptive transfer experiments; donor mice (Ly5.1 C57Bl6 congenic mice) were immunized with 50μg of KAS2-A1 with 3.8 ISCO™ units of ISCOMATRIX™ adjuvant (CSL Behring LLC, King of Prussia, PA; ISCOMATRIX is a registered trademark of ISCOTEC Ab a CSL company; ISCO is a registered trademark of CSL) per mouse or 50μg/mouse low endotoxin chicken albumin (OVA, Hyglos) (day 0) and IgG1 expressing B cells; IgG2 expressing B cells and CD4+ T cells isolated (day 30) from spleen and lymph nodes (inguinal, popliteal and submandibular) using magnetic bead separation; CD4+ T cells were isolated using anti-CD4 beads and IgG1 expressing B cells and IgG2 expressing B cells were using anti-IgG1/IgG2 B beads andAutoMACS cell sorter (Miltenyi Biotec, Bergisch Gladbach, Germany) as per the manufacturer’s instructions. Isolated cells were resuspended in PBS containing 0.5 μg of KAS2-A1/200μL or 0.5 μg of OVA/200μL, respectively, to aid *in vivo* cell recovery and activation and immediately transferred into recipient mice. Recipient mice (Ly5.2 C57Bl6 congenic mice) were injected (day 30) i.v. with 1 x 10^6^ cells; IgG1 or IgG2 expressing B cells or CD4+ T cells from KAS2-A1 immunized mice or B cells from ovalbumin immunized mice to act as an antigen-specific B cell control. The recipient mice were pre-treated with antibiotics and were orally challenged from day 31 with a total of four doses (days 31, 33, 35, 37 as described above) of 1.0 x 10^10^ total cells of *P. gingivalis* W50 or treated with PG buffer containing 2 % CMC alone (non-challenged group as N-C). Mice were killed 62 days from the first oral challenge and alveolar bone loss determined and bio-assays preformed as described above.

For the KAS2-A1-pAb therapeutic treatment experiments; BALB/c mice were pre-treated with antibiotics and for two groups purified KAS2-A1 rabbit polyclonal antibodies (pAb) in PBS or non-specific pAb in PBS (PBS/Alum immunized rabbits) were injected (i.p. 500μg/mouse, day -1) and then intra-orally inoculated with 4 x doses of *P. gingivalis* (Day 0, 2, 4, 6). For a further two groups; BALB/c mice were pre-treated with antibiotics and purified KAS2-A1 rabbit polyclonal antibodies (pAb) in PBS or non-specific pAb in PBS applied intra-orally (topical, 2 x 250μg/application) 15 mins pre- and post- each oral challenge 4 x doses of 1.0 x 10^10^ total cells of *P. gingivalis* in PG buffer containing 2% CMC (Day 0, 2, 4, 6). Other groups of BALB/c mice were treated with amoxicillin (500µg/mL drinking water, Day 0) or were not infected (non-challenged, N-C) mice. Mice were killed 62 days from the first oral inoculation and alveolar bone loss determined and bio-assays preformed as described above.

**Measurement of alveolar bone loss in mouse maxillae**

Maxillae to be examined for bone loss were boiled (1 min) in deionized water, mechanically defleshed, and immersed in 2% w/v potassium hydroxide (16 h, 25°C). Maxillae were washed twice with deionized water (25 °C), dried (1 h, 37 °C) and stained with 0.5% w/v aquoues methylene blue. Digital images of the buccal side of the maxillae were captured with an Olympus DP12 digital camera mounted on a dissecting microscope, using OLYSIA BioReport software version 3.2 (Olympus Australia Pty Ltd, New South Wales, Australia) to assess horizontal bone loss. Maxillae were oriented so that the buccal and lingual molar cusps were superimposed. Images were captured with a micrometre in frame, so that measurements could be standardized for each image. Horizontal bone loss was defined as the loss occurring in a horizontal plane, perpendicular to the alveolar bone crest that resulted in a reduction of the crest height. The visible area from the cemento-enamel junction (CEJ) to the alveolar bone crest (ABC) for each molar was measured using OLYSIA BioReport software version 3.2 imaging software to give the total visible CEJ-ABC area in mm^2^. *P. gingivalis*-induced alveolar bone loss in mm^2^ was calculated by subtracting the total visible CEJ-ABC area of the uninoculated (N-C) group from the total visible CEJ-ABC area of each experimental group. Alveolar bone loss measurements were determined twice in a random and blinded protocol. Data are expressed as the mean ± standard deviation in mm^2^ and were analyzed using a one-way ANOVA and Dunnetts T3 post-hoc test.

**Determination of subclass antibody in sera using Enzyme-Linked Immunosorbent Assay (ELISA)**

ELISAs were performed to evaluate subclass antibody in sera as described in Pathirana *et al.*[^11^](#_ENREF_11) in triplicate using a solution (5 μg/mL) of either the RgpA-Kgp complexes or FK W50 cells in 0.1M PBS (pH 7.4) to coat wells (16 h, 4 ºC) of flat-bottom polyvinyl microtiter plates (Microtiter; Dynatech Laboratories, McLean, VA, US). In these experiments the following antibody dilutions were used; a dilution of 1/2000 dilution of goat anti-mouse; IgG (M8642), IgG1 (M8770), IgG2a (M4434), IgG2b (M8067), IgG3 (M8270) and IgM (M4540) antibodies (Sigma, New South Wales, Australia) were used and a 1/8000 dilution of goat anti-mouse IgG2c antibody (1079-01; Southern Biotech, Birmingham, U.S.A.) was used in replacement of IgG2a antibody in the analysis of sera from C57BL6 mice[^12-14^](#_ENREF_12). A 1/4000 dilution of horseradish peroxidase-conjugated rabbit anti-goat IgG antibody (M5420; Sigma, New South Wales, Australia) was used to develop ELISA experiment.

**Determination of *P. gingivalis* colonization using real-time PCR**

Halved maxillae obtained at the termination of the mouse periodontitis experiments for real-time PCR analysis were immediately placed into lysis buffer and genomic DNA was isolated using the Promega Wizard Genomic DNA Kit. The animal tissue isolation protocol was followed as described by the manufacturer (Promega New South Wales, Australia) with the following modifications; maxillae tissues were digested in proteinase K (16 h, 55 °C) followed by a treatment using 3 mm tungsten carbide beads at 30 Hz for 45 s and DNA then eluted in 100 μL elution buffer.

The amount and quality of DNA present in the samples was determined using spectrometer readings (A_260_ and A_260/280_, respectively) and the Quant-iT DNA assay kit (Molecular Probes, Invitrogen, Mt. Waverly, Victoria, Australia) according to the manufacturer’s instructions with fluorescence measured on a Wallac 1420 multilabel counter (Perkin-Elmer, Wettesley, MA). DNA concentration (ng/μL) was then converted to the equivalent number of cells/μL for use as real-time PCR standards using chromosome sizes obtained from the bioinformatics resource for oral pathogens (www.brop.org)[^15^](#_ENREF_15). Molecular weight determined by assuming 1 base pair of dsDNA has a molecular weight of 649 Da[^16^](#_ENREF_16). The chromosomes size (base pairs) and the molecular weight of 1 genome (g) of *P. gingivalis* species are reported as 2,343,379 base pairs and 2.52 x 10^-15^ g. Each maxilla sample was diluted to contain 3 µg/μL DNA, and analyzed by real-time PCR. A *P. gingivalis* standard curve was created using known concentrations of *P. gingivalis* DNA (ten fold serial dilutions from 44 x 10^6^ to 44 x 10^1^ cells) added to maxilla DNA from an uninoculated mouse diluted to 10 ng/μL.

The cell numbers of *P. gingivalis* and total oral bacteria per maxilla were quantified using *P. gingivalis*-specific and universal primers. The universal and *P. gingivalis* primers were specific for the 16S rRNA gene (Life Technologies Australia Pty. Ltd., Victoria, Australia). The universal forward primer sequence was 5’-tcctacgggaggcagcagt, the reverse primer sequence was 5’-ggactaccagggtatctaatcctgtt and the probe sequence was 5’-/56-FAM/cgtattacc/ZEN/gcggctgctggcac/3IABkFQ/-3’[^17^](#_ENREF_17). The *P. gingivalis* forward primer sequence was 5’-tgcaacttgccttacagaggg, the reverse primer sequence was 5’-tcagttcccctacccatcgt and the probe sequence was 5’-/56-FAM/agctgtaag/ZEN/ataggcatgcgtcccattagctt/3IABkFQ/-3’[^18^](#_ENREF_18). The *P. gingivalis* reverse primer was modified to yield a shorter PCR product and to increase specificity for the *P. gingivalis* W50 strain. The probe sequences were modified to incorporate internal quencher technology.

Real-time PCR was performed using a Rotor-Gene 3000 system (Corbett Research, Australia) using a two step procedure. The first step consisted of a 40 µL reaction mixture containing 10 µL of the extracted DNA (equating to 3000 ng maxilla DNA), 25 µL of Taqman Fast Advanced Master-Mix (Life Technologies Australia Pty. Ltd., Victoria, Australia), 100 nM final concentration of Taqman probe (universal or *P. gingivalis*) (Life Technologies Australia Pty. Ltd., Victoria, Australia), and primers at a final concentration 100 nM (for the 10 cycle pre-PCR). For the second step a total of 10 µL reaction mixture was added consisting of 5 µL of Taqman Fast Advanced Master-Mix, 300 nM of forward and reverse primer and 50 nM probe. Each reaction mixture was dispensed using a CAS 1200 liquid-handling robot system (Corbett Research, New South Wales, Australia), and DNA samples added manually. The following PCR cycling conditions were used for the first step 10 cycle PCR: an initial hold (50 °C, 2 min), followed by a denaturation step (95 °C, 10 min). The following cycling conditions were repeated for 10 cycles: denaturation (95 °C, 20 s) and annealing/extension (60 °C, 60 s). The second step, 40 cycle PCR was as for the first step without the initial hold and with the denaturation/annealing repeated 40 times. Fluorescence data were collected immediately following the extension step of each cycle. The detection limit for the assay using the standard curve was determined to be equivalent to 40 *P. gingivalis* cells.

**Reagents used in flow cytometry and cell culture assays**

Antibodies to the following mouse antigens (Ag) were purchased from; BD Biosciences (Franklin Lakes, USA) CD4-APC conjugate (clone RM4-5), eBioscience (San Diego, CA, USA) TCRβ-FITC conjugate (clone H57-597), CD25-PE conjugate (clone PC61.5), CD69-PECy7 conjugate (clone MEL-14). All cell culture reagents were obtained from Sigma-Aldrich Pty. Ltd. (New South Wales, Australia) unless otherwise specified.

**Determination of cellular phenotypes in gingival tissues from mice orally challenged with *P. gingvalis* W50**

At the end of the experiments maxillae were harvested as described above (with gingival tissue still attached) and half maxillae were placed in digestion buffer [1 mg/mL collagenase II, 1 mg/mL collagenase IV and 25 Units DNase 1 in complete Dulbecco’s Modified Eagle Medium (DMEM) supplemented with 10% v/v heat-inactivated FBS (56 ºC, 30 min), 2 mM L-glutamine, 2 mM sodium pyruvate, 0.1mM 2-Me, 30 g/mL gentamicin and 100 IU/mL penicillin] (37 ºC, 1 h). Bones were then removed out from the digestion and red blood cells were removed from the cell suspension by treatment with red blood cell lysis buffer (Sigma-Aldrich Pty. Ltd., New South Wales, Australia) (RT, 2 min) and then washed twice in Dulbecco’s PBS (800 *g*, 5 min). All cell suspensions and antibody solutions (1:200 unless otherwise stated) were resuspended in fluorescence activated cell sorting buffer (FACS buffer) (24% w/v BSA, 0.1% w/v sodium-Azide, 20mM EDTA in Dulbecco’s PBS) and all incubations performed on ice for 20 min. Cells were resuspended at 1.0 × 10^5^ cells/180 μL in FACS buffer in duplicate and incubated with 1:400 Fc Block (BD Pharmingen, San Diego, CA, USA). Cells were washed (800 *g*, 5 min) and stained with antibodies TCRβ–FITC, CD25-PE, CD4-APC and CD69-PE Cy7 (1:500) for surface cellular phenotypic analysis. Appropriate isotype controls were used in all cases. Cells were washed twice (800 *g*, 5 min) and resuspended in FACS buffer. For flow cytometric analysis, a typical forward and side-scatter gate was set to exclude dead cells and aggregates; a total of 1 × 10^4^ events in the gate were collected and analyzed using FC500 flow cytometer and Kaluza Flow Cytometry software V1.1 (Beckman Coulter Australia Pty. Ltd., New South Wales, Australia). The phenotypic analysis was carried out on lymphocyte population gated using forward and side scatter parameters followed by positive gating identification of CD4+ T cells (TCRβ and CD4 markers), activated CD4+ T cells (TCRβ, CD4 and CD69 markers), memory/activated CD4+ T cells (TCRβ, CD4 and CD25 markers).

**ELISPOT assays**

ELISPOT assay was performed using the MabTech ELIPSpot^Plus^ Kits for IL-4, IL-17 and IFNγ (resolvingIMAGES, Vic, Australia) as per the manufactures instructions. Lymph nodes and spleens were collected in enriched Dulbecco’s Modified Eagles Medium/Ham’s Nutrient Mixture F12 (DMEM) supplemented with 10% (v/v) heat inactivated (56ºC, 30 mins) foetal bovine serum (FBS), 2 mM glutamine, 2 mM sodium pyruvate, 0.1 mM 2-mercaptoethanol, 30 μg/mL gentamicin, 100 I.U./mL penicillin and 100 μg/mL streptomycin (JRH Biosciences, Parkville, Australia), L-arginine (116 mg/mL), L-asparagine (36 mg/mL), and folic acid (6 mg/mL, Sigma, Melbourne, Australia) and single cell suspensions made by passing the lymph nodes or spleens through a wire mesh (200 μm). Gingival tissue cells were prepared as described above and resuspended in DMEM.

Red blood cells were removed from the spleen cell suspension by treatment with ammonium tris chloride buffer (17 mM Tris-HCl, 140 mM ammonium chloride in MilliQ water, pH 7.2) for two minutes on ice, and then washed three times in enriched DMEM (500 g, 5 mins at room temperature, IEC Centra GP8R refrigerated centrifuge, Thermo Electron Corporation, Melbourne, Victoria, Australia.).

Monocytes and dead cells were removed from the lymph node cell suspension using Lympholyte®-M (Cedarlane® Laboratories Limited, Ontario, Canada) as per the manufacturers’ instructions. Lymph node T-cells were separated using mouse CD 90 (Thy1.2) magnetic micro beads (Miltenyi Biotech, Germany) and the AutoMACs (Miltenyi Biotech, Germany) as per the manufacturers’ instructions. T cells or gingival tissue cells (3 x 10^5^/well) were incubated with γ-radiated (2200 Rads) syngeneic spleen cells as a source of APCs (3 x 10^5^cells/well) and antigen FK-*P. gingivalis* W50 (5 μg/well). Plates were incubated at 37ºC in an atmosphere of 5% CO_2_ in air for 48 hours in a humidified incubator. The plates were then washed and developed as per the manufacture’s instructions and spots were allowed to develop for 20 – 30 minutes, before stopping the reaction by washing with water. The spots were counted using EliSpot Reader Lite (version 2.9. Autoimmun Diagnostika GmbH Ebinger Strasse 4, Strassberg, Germany). Data is expressed as spot forming cells per million (SFC/million) and statistically analyzed using one-way ANOVA and Dunnett’s 3T test (SPSS for Windows, Release 6.0; SPSS).

**Epitope analysis of the Kgp proteinase active site peptide (KAS-2) sequence**

The antibody binding sites for the Lys-specific proteinase active site peptide KAS2 (433-468) was determined by synthesizing N-terminally biotinylated overlapping eight residue peptides (offset by one, overlapping by seven residues) on a multipin peptide synthesis system (Chiron Technologies, Melbourne, Australia) using standard solid-phase peptide synthesis protocols for Fmoc chemistry. Biotinylated peptides (5µg/mL) in 0.1 M PBS, pH 7.4 were bound to strepavidin coated plates, overnight at 4°C (Nunc, NSW Australia). After the wells were washed four times with PBST epitope mapping of the plate-bound peptides was carried out by ELISA as per Chiron Technologies instructions using mouse sera at a dilution of 1:1000 in 1% w/v non-fat skim milk powder in 0.1 M PBS, pH 7.4, containing 0.1% v/v Tween 20 (SK-PBST). After the wells were washed six times with PBST, a 1/2,000 dilution of goat IgG to mouse IgG (Sigma, New South Wales, Australia) was added in SK-PBST and allowed to bind for 2 h at room temperature. Plates were washed six times in PBST, and a 1/5,000 dilution of horseradish peroxidase-conjugated rabbit anti-goat immunoglobulin (Sigma, New South Wales, Australia) in SK-PBST was added to each well and incubated for 1 h at room temperature. After the wells were washed six times with PBST, bound antibody was detected by the addition of 100 µl of ABTS substrate [0.9 mM 2,2'-azino-bis(3-ethylbenz-thiazoline-6) sulfonic acid in 80 mM citric acid containing 0.005% (vol/vol) hydrogen peroxide, pH 4.0] to each well. The optical density at 415 nm was measured using a microplate reader (Bio-Rad microplate reader, model 450).

**Determination of subclass antibody by an ELISA**

To determine the subclass antibody responses of mouse sera, enzyme-linked immunosorbent assays (ELISAs) were performed in triplicate using a either a 5 µg/mL solution of formalin killed *P. gingivalis* W50, 10 µg/mL solution of RgpA-Kgp complex, 10 µg/mL solution of KAS2 peptide, 10 µg/mL solution of recombinant A1 (rA1 751-1056) or 10 µg/mL solution of KAS2-A1 in phosphate-buffered saline (PBS) (0.01 M Na_2_HPO_4_, 1.5 mM KH_2_PO_4_, 0.15 M NaCl), pH 7.0, containing 0.1% (vol/vol) Tween 20 (PBST) to coat wells of flat-bottom polyvinyl microtiter plates (Dynatech Laboratories, McLean, VA). After removal of the coating solution, PBST containing 2% (wt/vol) skim milk powder was added to wells to block the uncoated plastic for 1 h at room temperature. After the wells were washed four times with PBST, serial dilutions of mouse sera in PBST containing 0.5% (wt/vol) skim milk (SK-PBST) were added to each well and incubated for 16 h at room temperature. After the wells were washed six times with PBST, a 1/2,000 dilution of goat IgG to mouse IgM, IgA, IgG1, IgG2a, IgG2b, or IgG3 (Sigma, New South Wales, Australia) was added in SK-PBST and allowed to bind for 2 h at room temperature. Plates were washed six times in PBST, and a 1/5,000 dilution of horseradish peroxidase-conjugated rabbit anti-goat immunoglobulin (Sigma, New South Wales, Australia) in SK-PBST was added to each well and incubated for 1 h at room temperature. After the wells were washed six times with PBST, bound antibody was detected by the addition of 100 µl of ABTS substrate [0.9 mM 2,2'-azino-bis(3-ethylbenz-thiazoline-6) sulfonic acid in 80 mM citric acid containing 0.005% (vol/vol) hydrogen peroxide, pH 4.0] to each well. The optical density at 415 nm was measured using a microplate reader (Bio-Rad microplate reader, model 450).

**Fluorescent labelling of bacteria**

The bacteria were fluorescently labelled by resuspension at a bacterial cell density of 3.5 × 10^9^ cells/mL in 0.5 g/L sodium bicarbonate (Sigma-Aldrich) containing the desired fluorochrome and incubation for 45 min at 37°C under gentle agitation. To fluorescently label bacteria, FITC (fluorescein-5-isothiocyanate, Invitrogen) or Alexa Fluor® 555 (AF-555, carboxylic acid succinimidyl ester, Invitrogen) were used at 10 μg/mL in 0.5 g/L sodium bicarbonate, and Alexa Fluor® 647 (AF-647, carboxylic acid succinimidyl ester, Invitrogen) was used at 150 μg/mL in 0.5 g/L sodium bicarbonate to give equi-fluorescence/bacteria. After incubation with the fluorochromes, bacteria were pelleted by centrifugation at 7,000 *g* for 10 min at 4°C, washed three times in phosphate buffered saline (PBS, pH 7.4) to remove the unbound fluorochromes, and then resuspended in EMEM (unsupplemented) and used in binding and co-aggregation assays.

**Bacterial co-aggregation assay**

For flow cytometric analysis, *P. gingivalis* and *T. denticola* were labelled with Alexa Fluor® succinimidyl ester 555 (AF-555) and AF-647 (Invitrogen) respectively, and fluorescence expressed as % per quadrant. Bacteria were harvested and fluorescently labelled as described above and resuspended at 5 x 10^7^ cell/mL. Two hundred microliters of *P. gingivalis* suspension was pre-incubated (60 mins) with purified KAS2-A1 pAb or non-specific (NS) pAb (250 μg/mL) and then incubated (60 mins) with AlexaFluor 647-labelled *T. denticola* cells (5 x 10^7^/mL). Flowing incubation a 1 mM solution of SYTO 9 was added to form a final reaction solution dilution of 1:1000. The bacterial co-aggregation was determined by gating on SYTO 9 positive events (all viable bacteria), and within this gated population, *T. denticola* cells were identified by FL4 fluorescence, *P. gingivalis* cells by FL2 fluorescence, and co-aggregated bacteria as positive for both FL4 and FL2 fluorescence measured as AF-555 and AF-647 double positives cytometrically as described above in triplicate.

**Bacterial binding to oral epithelial cells**

For oral epithelial cell (OKF6-TERT2[^19^](#_ENREF_19)) binding studies, bacteria were grown as described above, harvested by centrifugation (7,000 *g*, 20 min, 4°C) and washed once with PBS, based on the methods of Pathirana *et al.*[^20^](#_ENREF_20).

Oral epithelial cells were grown to 95% confluence in 24-well polystyrene cell culture plates and media removed prior addition of *P. gingivalis* cells. FITC-labelled *P. gingivalis* W50 cells (1 x 10^8^/mL) in EMEM media (unsuplemented) were pre-incubated (60 mins) with purified KAS2-A1 pAb or non-specific (NS) pAb (100 μg/mL) and then incubated (90 mins) with a confluent monolayer of oral epithelial cells at a bacteria to cell ratio of 20:1 andincubated (45 min, 37°C, 5% CO_2_).

After incubation the supernatant containing bacteria and bacterial protease-displaced epithelial cells were removed, and the remaining oral epithelial cells were collected by treating with trypsin (0.25% Trypsin-EDTA Solution, Sigma-Aldrich) for 5 min at 37°C. The trypsin-treated oral epithelial cells were then collected and added to the corresponding collected supernatant, and the oral epithelial cells were then pelleted by centrifugation (800 *g*, 5 min). The oral epithelial cells were washed twice in PBS and resuspended in PBS for analysis by flow cytometry.

Oral epithelial cells were identified by size (forward scatter) and CD29-PE fluorescence via a 575 nm filter, and gated to exclude unbound bacteria and cellular debris. At least 10,000 oral epithelial cells were identified and analyzed for the adherence of FITC-labelled *P. gingivalis*. All multi-parametric data were analyzed by MXP Cytometry List Mode Data Acquisition and Analysis Software (Beckman Coulter, NSW, Australia). All measurements were performed in triplicate and statistically analyzed by a paired, two-tailed Student’s t-Test.

**Inhibition of RgpA-Kgp complex binding to host proteins**

Inhibition of RgpA-Kgp complex binding was performed as previously described[^10^](#_ENREF_10). Briefly, RgpA-Kgp complex (0.5 μg/mL) was incubated (3 hr) with fibronectin, fibrinogen and haemoglobin pre-coated ELISA plates in the presence of KAS2-A1 pAb or non-specific (NS) pAb (5 μg/mL) in 0.1M PBS containing 1 mM TLCK. The percent of inhibition of binding was determined from the binding of RgpA-Kgp complex in the absence of antibody. Data expressed as the mean ± standard deviation of the percent inhibition and were analyzed using a Student’s T test. * indicates data that are significantly different (*p* < 0.01) from the data for the NS-pAb group.

**Inhibition of Lys-X and Arg-X proteinase activity of *P. gingivalis* whole cells**

*P. gingivalis* W50 whole cells (1 x 10^8^/mL) in TC150 buffer containing 2 mM L-cysteine was pre-incubated (0.5, 4 and 24 hours) with purified KAS2-A1 pAb or non-specific (NS) pAb (100 μg/mL) and whole cell Arg-X and Lys-X proteinase activity determined as previously described[^11^](#_ENREF_11). Data is expressed as the mean ± standard deviation of the percent inhibition of *P. gingivalis* W50 whole cells incubated for equivalent times in the absence of antibodies and were analyzed using a Student’s T test. * indicates data that are significantly different (*p* < 0.01) from the data for the NS-pAb group

**REFERENCES**

1. McKee, A.S. *et al.* Effect of hemin on the physiology and virulence of *Bacteroides gingivalis* W50. *Infect. Immun.* **52**, 349-355 (1986).

2. Slots, J. Importance of black-pigmented *Bacteriodes* in human periodontal disease, in *Host-Parasite Interaction in Periodontal Disease*. (ed. R.J. Genco, Merganhagan, S.E.) 27-45 (American Society for Microbiology, Washington D.C.; 1982).

3. Makinen, K.K., Chen, C.Y. & Makinen, P.L. Proline iminopeptidase from the outer cell envelope of the human oral spirochete *Treponema denticola* ATCC 35405. *Infect. Immun.* **64**, 702-708. (1996).

4. Leschine, S.B. & Canale-Parola, E. Rifampin as a selective agent for isolation of oral spirochetes. *J. Clin. Microbiol.* **12**, 792-795. (1980).

5. Kesavalu, L., Walker, S.G., Holt, S.C., Crawley, R.R. & Ebersole, J.L. Virulence characteristics of oral treponemes in a murine model. *Infect. Immun.* **65**, 5096-5102. (1997).

6. Veith, P.D. *et al.* Outer membrane proteome and antigens of *Tannerella forsythia*. *J. Proteome Res.* **8**, 4279-4292 (2009).

7. Pathirana, R.D., O'Brien-Simpson, N.M., Veith, P.D., Riley, P.F. & Reynolds, E.C. Characterization of proteinase-adhesin complexes of *Porphyromonas gingivalis*. *Microbiology (SGM)* **152**, 2381-2394 (2006).

8. Frazer, L.T. *et al.* Vaccination with recombinant adhesins from the RgpA-Kgp proteinase-adhesin complex protects against *Porphyromonas gingivalis* infection. *Vaccine* **24**, 6542-6554 (2006).

9. Baker, P.J., Evans, R.T. & Roopenian, D.C. Oral infection with *Porphyromonas gingivalis* induces alveolar bone loss in immunocompetent and severe combined immunodeficient mice. *Arch. Oral Biol.* **39**, 1035-1040 (1994).

10. O'Brien-Simpson, N.M. *et al.* An immune response directed to proteinase and adhesin functional epitopes protects against *Porphyromonas gingivalis*-induced bone loss. *J. Immunol.* **175**, 3980-3989 (2005).

11. Pathirana, R.D., O'Brien-Simpson, N.M., Brammar, G.C., Slakeski, N. & Reynolds, E.C. Kgp and RgpB, but not RgpA, are important for *Porphyromonas gingivalis* virulence in the murine periodontitis model. *Infect. Immun.* **75**, 1436-1442 (2007).

12. Jouvin-Marche, E. *et al.* The mouse Igh-1a and Igh-1b H chain constant regions are derived from two distinct isotypic genes. *Immunogenetics* **29**, 92-97 (1989).

13. Martin, F., Won, W.J. & Kearney, J.F. Generation of the germline peripheral B cell repertoire: VH81X-lambda B cells are unable to complete all developmental programs. *J. Immunol.* **160**, 3748-3758 (1998).

14. Morgado, M.G., Cam, P., Gris-Liebe, C., Cazenave, P.A. & Jouvin-Marche, E. Further evidence that BALB/c and C57BL/6 gamma 2a genes originate from two distinct isotypes. *EMBO J.* **8**, 3245-3251 (1989).

15. Chen, T., Abbey, K., Deng, W.J. & Cheng, M.C. The bioinformatics resource for oral pathogens. *Nucleic Acids Res.* **33 (Web Server issue)**, W734-740 (2005).

16. Ausubel, F.M. *et al.* *Current Protocols in Molecular Biology*, Vol. 1. (Greene and Wiley-Interscience, New York; 1995).

17. Nadkarni, M.A., Martin, F.E., Jacques, N.A. & Hunter, N. Determination of bacterial load by real-time PCR using a broad-range (universal) probe and primers set. *Microbiology* **148**, 257-266 (2002).

18. Gaetti-Jardim, E., Jr., Marcelino, S.L., Feitosa, A.C., Romito, G.A. & Avila-Campos, M.J. Quantitative detection of periodontopathic bacteria in atherosclerotic plaques from coronary arteries. *J. Med. Microbiol.* **58**, 1568-1575 (2009).

19. Dickson, M.A. *et al.* Human keratinocytes that express hTERT and also bypass a p16(INK4a)-enforced mechanism that limits life span become immortal yet retain normal growth and differentiation characteristics. *Mol. Cell. Biol.* **20**, 1436-1447 (2000).

20. Pathirana, R.D., O'Brien-Simpson, N.M., Visvanathan, K., Hamilton, J.A. & Reynolds, E.C. Flow cytometric analysis of adherence of *Porphyromonas gingivalis* to oral epithelial cells. *Infect. Immun.* **75**, 2484-2492 (2007).

**Supplementary Figures**


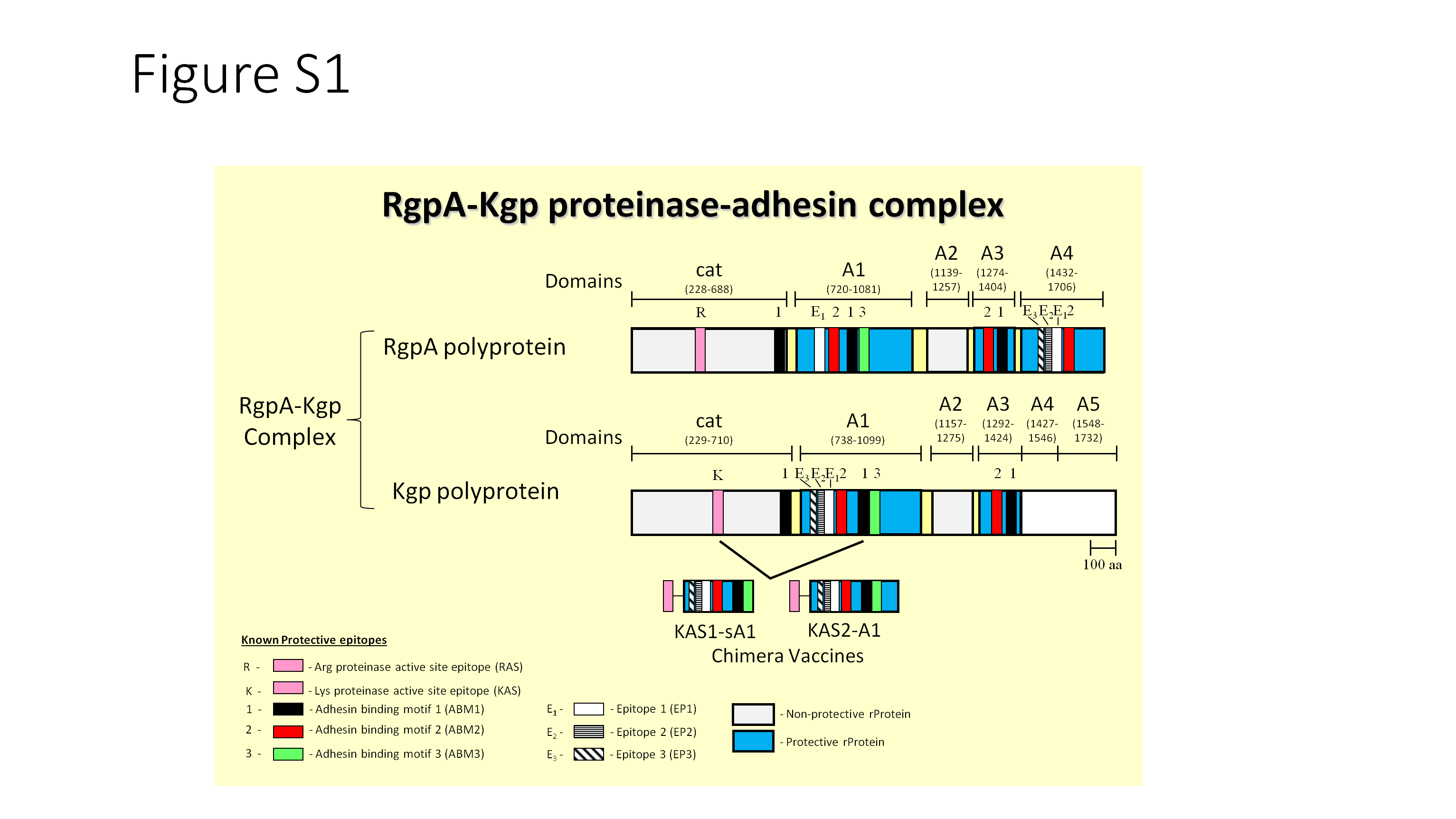


**Supplementary Figure 1.** **Schematic representation of the RgpA-Kgp proteinase adhesin complex**. The schematic shows the proteinase (cat) and adhesin (A1-4; A1-5) domains[^1^](#_ENREF_1), the KAS1-sA1 and KAS2-A1 chimera and the relative positions of protective peptide and protein sequences. Adapted from O’Brien-Simpson *et al*^2^.

**References**

1. Pathirana, R.D., O'Brien-Simpson, N.M., Veith, P.D., Riley, P.F. & Reynolds, E.C. Characterization of proteinase-adhesin complexes of *Porphyromonas gingivalis*. *Microbiology (SGM)* **152**, 2381-2394 (2006).

2. O'Brien-Simpson, N.M. et al. An immune response directed to proteinase and adhesin functional epitopes protects against *Porphyromonas gingivalis*-induced bone loss. *J. Immunol*. **175**, 3980-3989 (2005).

**Supplementary Figure 2. Sequence and SDS-PAGE analysis of KAS1-sA1 and KAS2-A1.** (**a**) amino acid sequence (single letter code) of KAS1-sA1 and KAS2-A1, highlighted sequences show the immunogenic/protective epitopes; KAS1/2 (blue); ABM1 (grey); ABM2 (yellow); ABM3 (green); EP1 (dark green); EP2 (red) and EP3 (magenta). Italicized letters show the A1 sequence. The bold/underlined/italicized letters show the extended active site sequence and the extended adhesin sequence, respectively in the KAS2-A1 construct. (**b**) SDS-PAGE gel of purified recombinant proteins; lanes: 1, KAS2-A1; 2, extended A1; 3, A1 – minimal immunogenic sequence and 4, KAS1-sA1. Molecular mass markers (Pharmacia) are indicated in kDa. Each of the purified recombinant proteins consisted of one major protein band with molecular weights of 40, 36, 31 and 32 kDa corresponding to KAS2-A1, extended-A1, minimal immunogenic sequence-A1 and KAS1-sA1, respectively, which corresponded to the calculated molecular masses of each of the His-tag recombinant proteins using ProtParam. KAS1-sA1 represents the minimal immunogenic sequences of KAS (Kgp-cat, 432-454) and KgpA1 (759-989) and KAS2-A1 extends the minimal immunogenic sequences of KAS (Kgp-cat, 433-468) and KgpA1 (751- 1056). Amino acid sequence numbers based on *Porphyromonas gingivalis* lysine-specific cysteine proteinase (prtK) gene [Genbank accession number, U75366].

**Supplementary Figure 3. Characterization of the antigenicity of recombinant chimera proteins KAS1-sA1 and KAS2-A1.** (**a + b)** PEPSCAN analysis of peptide-specific antibody reactivity to overlapping peptides representing the KAS2 peptide sequence 433-NTGVSFANYTAHGSETAWADPLLTTSQLKALTNKDK-468. (**a**) KAS2 overlapping peptides (offset 1, overlap 7) probed with KAS1-sA1 (white bars), KAS2-A1 (black bars) antisera. (**b**) KAS2 overlapping peptides (offset 1, overlap 7) probed with KAS2 peptide-DT conjugate antisera. Each bar displays the antibody reactivity (optical density [OD] at 415 nm) representing the mean ± standard deviation of three values.

**Supplementary Figure 4. Flow cytometry dot plots of gingival and SMLN lymphocytes from the adoptive transfer experiments**. This figure shows the percent of plasma B cells [CD138^+^, CD19^+^] for each group. Data are representative of two independent experiments.

**Supplementary Figure 5.** **Characterization of the rabbit anti-KAS2-A1 purified polyclonal antibodies (pAb)**. (**a**) Antisera pAb-KAS2-A1 and pAb-PBS/Alum was used to probe formalin killed *P. gingivalis* strain W50 as the absorbed antigen in an ELISA and antibody responses are expressed as the absorbance at 405nm. (**b**) Western blot analysis using pAb KAS2-A1 antisera to probe; lanes: 1, *P. gingivalis* whole cell lysate; 2, RgpA-Kgp complex; 3, KAS2-A1; and 4, KAS1-sA1. Molecular mass markers (Pharmacia) are indicated in kDa.


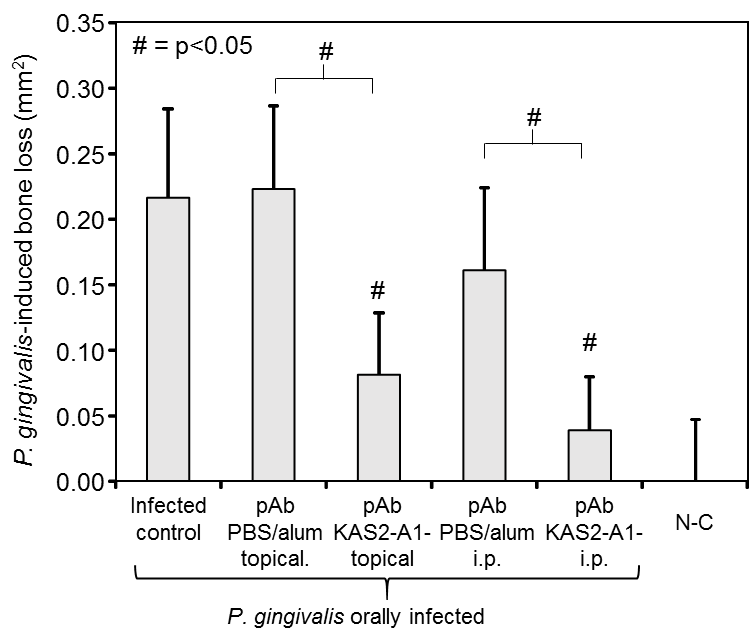


**Supplementary Figure 6. KAS2-A1 pAbs protect in a therapeutic treatment model of periodontitis.** Purified KAS2-A1 polyclonal antibodies administered by oral topical or intraperitoneal injection protect mice against *P. gingivalis*-induced bone loss in the periodontitis model. Purified KAS2-A1 rabbit polyclonal antibodies were injected (i.p. 500μg/mouse) on day 19 post oral challenge with *P. gingivalis* or applied intra-orally (topical, 4 x 500μg/application) from day 19 post oral challenge. Bacteria-induced bone resorption was determined as described in the Material and Methods section for each group (n = 12), and the data are expressed as the mean ± standard deviation in mm^2^ and were analyzed using a one-way ANOVA and Dunnetts T3 post-hoc test. # indicates data that are significantly different (*p* < 0.05) from the data for *P. gingivalis* challenged group.

**Supplementary Figure 7.** **KAS2-A1 pAb recognition of a panel of *P. gingivalis* laboratory strains and clinical isolates.** KAS2-A1-pAb, RgpA-Kgp complex-pAb and non-specific (NS)-pAb were used to probe membrane extracts of a panel of *P. gingivalis* laboratory strains; 381 (serotype A), A7A1-28 (serotype B), W50 (serotype C), ATCC 33277 (serotype D), ATCC 53978, ATCC 49417, YH522 and clinical isolates 84-3, RA, 3-3, 3A1, 7B-TORR and 15-9 that were used as the absorbed antigen in an ELISA. Data are expressed as the optical density (405nm) obtained minus double the background level, with each titre representing the mean ± standard deviation of three values.

**Supplementary Table**

**Supplementary Table 1. Oligonucleotide primers used for the amplification of the nucleotide sequences encoding the various fragments and chimeras of Kgp A1 and KAS**

| Recombinant protein | Primers | Characteristics* (5’-3’) |
| --- | --- | --- |
| KAS2  KAS2-FOR | 5’-GACCATGGCTCATCACCATCACCATCACA ATACCGGAGTCAGCTTTGCA-3’ | GA buffer-NcoI (including ATG start)-CT-(His)_6_-AS (nt 1992-2012) |
| KAS2-REV | 5’-GACTCGAGTTATTTGTCCTTATTAGTGAG TGCTTTC-3’) | GA buffer-XhoI-TTA Stop-KAS1 (nt 2099-2075) |
| A1  A1-FOR | 5’-GACCATGGCTTGGGGAGACAATACGGGT TAC-3’ | GA buffer-NcoI (including ATG start)-CT–A1 (nt 2946-2966) |
| A1-REV | 5’-GACTCGAGACCTCCGTTAGGCAAATCC-3’ | GA buffer-XhoI-A1 (nt 3863-3845) |
| KAS2-A1  KAS2-A1-REV | 5’-CCGTATTGTCTCCCCATTTGTCCTTATTAG TGAGTGCTTTC-3’ | A1 (nt 2961-2946)-KAS1 (nt 2099-2075) |
| KAS2-A1-FOR | 5’-CACTAATAAGGACAAATGGGGAGACAAT ACGGGTTAC-3’ | KAS1 (nt 2084-2099)-A1 (nt 2946-2966) |
| KAS1-sA1  KAS1-sA1-FOR1 | 5’-CATGGATCTGAGACCGCATGGGCTGATC CACTTTTCTTGTTGGATGCCGAT-3’ | AS (nt 2025-2057)-A1 (nt 2970-2987)- |
| KAS1-sA1-FOR2 | 5’-CCATGGCTTTGAATACCGGAGTCAGCTTT GCAAACTATACAGCGCATGGATCTGAGACCGCA-3’ | NcoI-CT-AS (nt 1989-2042) |
| KAS1-sA1-REV | 5’-CTCGAGGAATGATTCGGAAAGTGTT-3’ | XhoI-A1(nt 3663-3644) |

* nucleotide (nt) sequence numbers from lysine-specific cysteine proteinase gene sequence accession number U75366
