# Supplementary material for: A therapeutic Porphyromonas gingivalis gingipain vaccine induces neutralising IgG1 antibodies that protect against experimental periodontitis
Source: NPJ Vaccines. 2016 Dec 1;1:16022–. doi: 10.1038/npjvaccines.2016.22 (PMC5707886; doi:10.1038/npjvaccines.2016.22)
Supplement: Supplementary Information [file npjvaccines201622-s2.doc]

**Supplementary Figures and Tables**


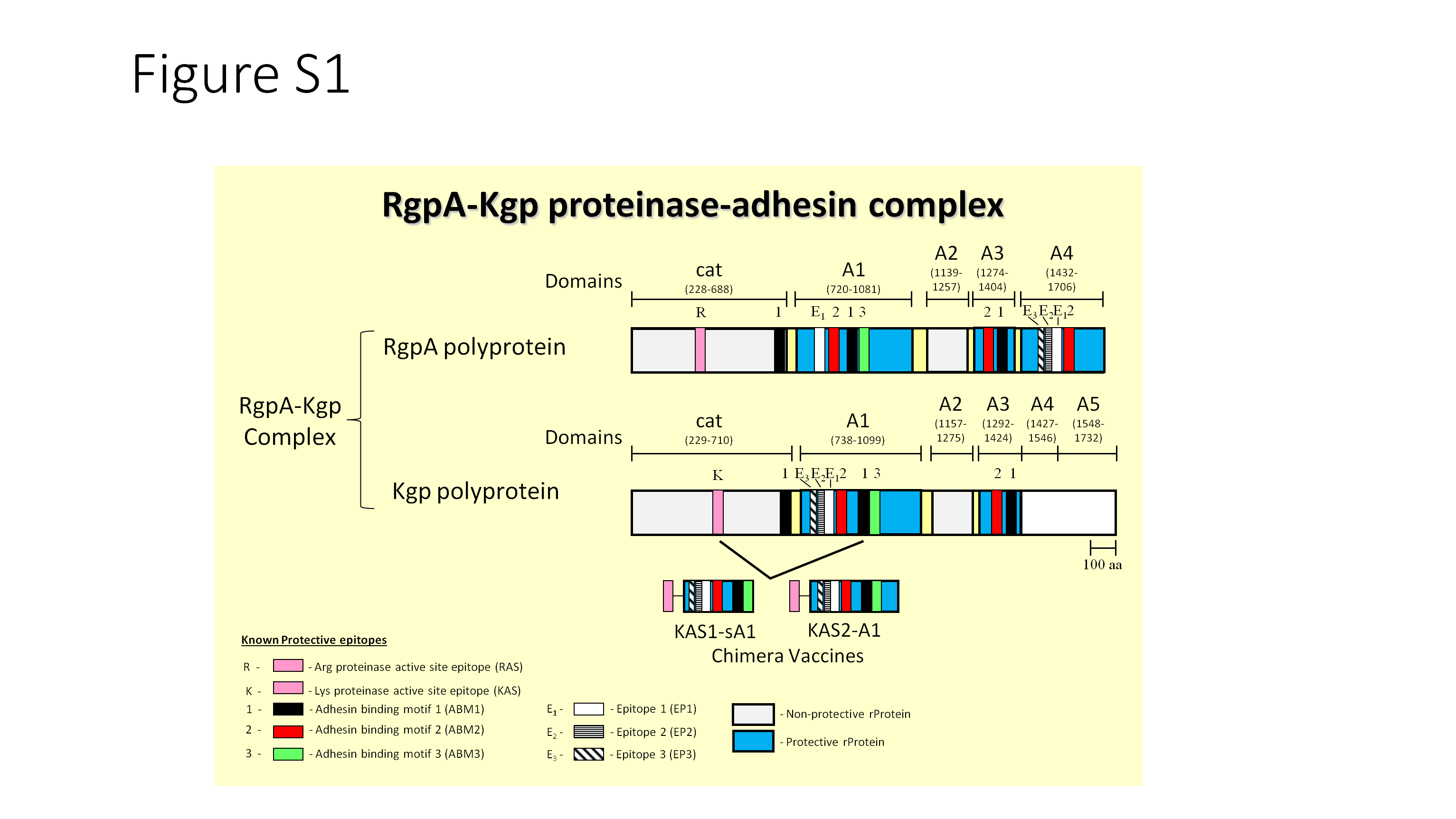


**Supplementary Figure 1.** **Schematic representation of the RgpA-Kgp proteinase adhesin complex**. The schematic shows the proteinase (cat) and adhesin (A1-4; A1-5) domains[^1^](#_ENREF_1), the KAS1-sA1 and KAS2-A1 chimera and the relative positions of protective peptide and protein sequences. Adapted from O’Brien-Simpson *et al*[^2^](#_ENREF_2).

**References**

1. Pathirana, R.D., O'Brien-Simpson, N.M., Veith, P.D., Riley, P.F. & Reynolds, E.C. Characterization of proteinase-adhesin complexes of *Porphyromonas gingivalis*. *Microbiology (SGM)* **152**, 2381-2394 (2006).

2. O'Brien-Simpson, N.M. *et al.* An immune response directed to proteinase and adhesin functional epitopes protects against *Porphyromonas gingivalis*-induced bone loss. *J. Immunol.* **175**, 3980-3989 (2005).

**Supplementary Figure 2. Sequence and SDS-PAGE analysis of KAS1-sA1 and KAS2-A1.** (**a**) amino acid sequence (single letter code) of KAS1-sA1 and KAS2-A1, highlighted sequences show the immunogenic/protective epitopes; KAS1/2 (blue); ABM1 (grey); ABM2 (yellow); ABM3 (green); EP1 (dark green); EP2 (red) and EP3 (magenta). Italicized letters show the A1 sequence. The bold/underlined/italicized letters show the extended active site sequence and the extended adhesin sequence, respectively in the KAS2-A1 construct. (**b**) SDS-PAGE gel of purified recombinant proteins; lanes: 1, KAS2-A1; 2, extended A1; 3, A1 – minimal immunogenic sequence and 4, KAS1-sA1. Molecular mass markers (Pharmacia) are indicated in kDa. Each of the purified recombinant proteins consisted of one major protein band with molecular weights of 40, 36, 31 and 32 kDa corresponding to KAS2-A1, extended-A1, minimal immunogenic sequence-A1 and KAS1-sA1, respectively, which corresponded to the calculated molecular masses of each of the His-tag recombinant proteins using ProtParam. KAS1-sA1 represents the minimal immunogenic sequences of KAS (Kgp-cat, 432-454) and KgpA1 (759-989) and KAS2-A1 extends the minimal immunogenic sequences of KAS (Kgp-cat, 433-468) and KgpA1 (751- 1056). Amino acid sequence numbers based on *Porphyromonas gingivalis* lysine-specific cysteine proteinase (prtK) gene [Genbank accession number, U75366].

**Supplementary Figure 3. Characterization of the antigenicity of recombinant chimera proteins KAS1-sA1 and KAS2-A1.** (**a + b)** PEPSCAN analysis of peptide-specific antibody reactivity to overlapping peptides representing the KAS2 peptide sequence 433-NTGVSFANYTAHGSETAWADPLLTTSQLKALTNKDK-468. (**a**) KAS2 overlapping peptides (offset 1, overlap 7) probed with KAS1-sA1 (white bars), KAS2-A1 (black bars) antisera. (**b**) KAS2 overlapping peptides (offset 1, overlap 7) probed with KAS2 peptide-DT conjugate antisera. Each bar displays the antibody reactivity (optical density [OD] at 415 nm) representing the mean ± standard deviation of three values.

**Supplementary Figure 4. Flow cytometry dot plots of gingival and SMLN lymphocytes from the adoptive transfer experiments**. This figure shows the percent of plasma B cells [CD138^+^, CD19^+^] for each group. Data are representative of two independent experiments.

**Supplementary Figure 5.** **Characterization of the rabbit anti-KAS2-A1 purified polyclonal antibodies (pAb)**. (**a**) Antisera pAb-KAS2-A1 and pAb-PBS/Alum was used to probe formalin killed *P. gingivalis* strain W50 as the absorbed antigen in an ELISA and antibody responses are expressed as the absorbance at 405nm. (**b**) Western blot analysis using pAb KAS2-A1 antisera to probe; lanes: 1, *P. gingivalis* whole cell lysate; 2, RgpA-Kgp complex; 3, KAS2-A1; and 4, KAS1-sA1. Molecular mass markers (Pharmacia) are indicated in kDa.


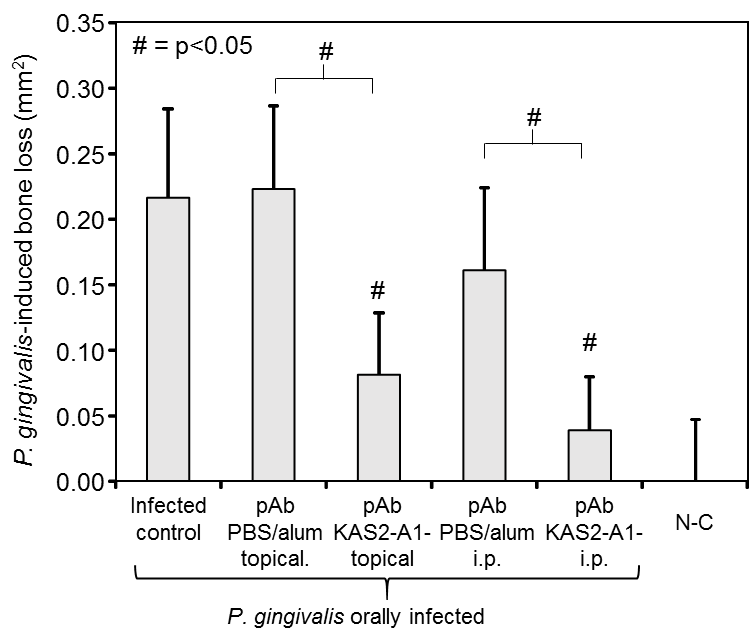


**Supplementary Figure 6. KAS2-A1 pAbs protect in a therapeutic treatment model of periodontitis.** Purified KAS2-A1 polyclonal antibodies administered by oral topical or intraperitoneal injection protect mice against *P. gingivalis*-induced bone loss in the periodontitis model. Purified KAS2-A1 rabbit polyclonal antibodies were injected (i.p. 500μg/mouse) on day 19 post oral challenge with *P. gingivalis* or applied intra-orally (topical, 4 x 500μg/application) from day 19 post oral challenge. Bacteria-induced bone resorption was determined as described in the Material and Methods section for each group (n = 12), and the data are expressed as the mean ± standard deviation in mm^2^ and were analyzed using a one-way ANOVA and Dunnetts T3 post-hoc test. # indicates data that are significantly different (*p* < 0.05) from the data for *P. gingivalis* challenged group.

**Supplementary Figure 7.** **KAS2-A1 pAb recognition of a panel of *P. gingivalis* laboratory strains and clinical isolates.** KAS2-A1-pAb, RgpA-Kgp complex-pAb and non-specific (NS)-pAb were used to probe membrane extracts of a panel of *P. gingivalis* laboratory strains; 381 (serotype A), A7A1-28 (serotype B), W50 (serotype C), ATCC 33277 (serotype D), ATCC 53978, ATCC 49417, YH522 and clinical isolates 84-3, RA, 3-3, 3A1, 7B-TORR and 15-9 that were used as the absorbed antigen in an ELISA. Data are expressed as the optical density (405nm) obtained minus double the background level, with each titre representing the mean ± standard deviation of three values.

**Supplementary Table 1. Oligonucleotide primers used for the amplification of the nucleotide sequences encoding the various fragments and chimeras of Kgp A1 and KAS**

| Recombinant protein | Primers | Characteristics* (5’-3’) |
| --- | --- | --- |
| KAS2  KAS2-FOR | 5’-GACCATGGCTCATCACCATCACCATCACA ATACCGGAGTCAGCTTTGCA-3’ | GA buffer-NcoI (including ATG start)-CT-(His)_6_-AS (nt 1992-2012) |
| KAS2-REV | 5’-GACTCGAGTTATTTGTCCTTATTAGTGAG TGCTTTC-3’) | GA buffer-XhoI-TTA Stop-KAS1 (nt 2099-2075) |
| A1  A1-FOR | 5’-GACCATGGCTTGGGGAGACAATACGGGT TAC-3’ | GA buffer-NcoI (including ATG start)-CT–A1 (nt 2946-2966) |
| A1-REV | 5’-GACTCGAGACCTCCGTTAGGCAAATCC-3’ | GA buffer-XhoI-A1 (nt 3863-3845) |
| KAS2-A1  KAS2-A1-REV | 5’-CCGTATTGTCTCCCCATTTGTCCTTATTAG TGAGTGCTTTC-3’ | A1 (nt 2961-2946)-KAS1 (nt 2099-2075) |
| KAS2-A1-FOR | 5’-CACTAATAAGGACAAATGGGGAGACAAT ACGGGTTAC-3’ | KAS1 (nt 2084-2099)-A1 (nt 2946-2966) |
| KAS1-sA1  KAS1-sA1-FOR1 | 5’-CATGGATCTGAGACCGCATGGGCTGATC CACTTTTCTTGTTGGATGCCGAT-3’ | AS (nt 2025-2057)-A1 (nt 2970-2987)- |
| KAS1-sA1-FOR2 | 5’-CCATGGCTTTGAATACCGGAGTCAGCTTT GCAAACTATACAGCGCATGGATCTGAGACCGCA-3’ | NcoI-CT-AS (nt 1989-2042) |
| KAS1-sA1-REV | 5’-CTCGAGGAATGATTCGGAAAGTGTT-3’ | XhoI-A1(nt 3663-3644) |

* nucleotide (nt) sequence numbers from lysine-specific cysteine proteinase gene sequence accession number U75366
